# WAX INDUCER1 (HvWIN1) transcription factor regulates free fatty acid biosynthetic genes to reinforce cuticle to resist Fusarium head blight in barley spikelets

**DOI:** 10.1093/jxb/erw187

**Published:** 2016-05-18

**Authors:** Arun Kumar, Kalenahalli N. Yogendra, Shailesh Karre, Ajjamada C. Kushalappa, Yves Dion, Thin M. Choo

**Affiliations:** ^1^Plant Science Department, McGill University, Sainte-Anne-de-Bellevue, QC H9X3V9, Canada; ^2^Centre de Recherché sur les Grains Inc., 740, chemin Trudeau, Saint-Mathieu-de-Beloeil, QC J3G0E2, Canada; ^3^Eastern Cereal and Oilseed Research Centre, Agriculture and Agri-Food Canada, 960 Carling Ave., Ottawa, ON K1A0C6, Canada

**Keywords:** Cuticle reinforcement, free fatty acids, Fusarium head blight, quantitative resistance, resistance-related metabolites, transcription factors.

## Abstract

Fusarium head blight (FHB), caused by *Fusarium graminearum,* is one of the most devastating diseases of wheat and barley. Resistance to FHB is highly complex and quantitative in nature, and is most often classified as resistance to spikelet infection and resistance to spread of pathogen through the rachis. In the present study, a resistant (CI9831) and a susceptible (H106-371) two-row barley genotypes, with contrasting levels of spikelet resistance to FHB, pathogen or mock-inoculated, were profiled for metabolites based on liquid chromatography and high resolution mass spectrometry. The key resistance-related (RR) metabolites belonging to fatty acids, phenylpropanoids, flavonoids and terpenoid biosynthetic pathways were identified. The free fatty acids (FFAs) linoleic and palmitic acids were among the highest fold change RR induced (RRI) metabolites. These FFAs are deposited as cutin monomers and oligomers to reinforce the cuticle, which acts as a barrier to pathogen entry. Quantitative real-time PCR studies revealed higher expressions of *KAS2*, *CYP86A2*, *CYP89A2*, *LACS2* and *WAX INDUCER1* (*HvWIN1*) transcription factor in the pathogen-inoculated resistant genotype than in the susceptible genotype. Knockdown of *HvWIN1* by virus-induced genes silencing (VIGS) in resistant genotype upon pathogen inoculation increased the disease severity and fungal biomass, and decreased the abundance of FFAs like linoleic and palmitic acids. Notably, the expression of *CYP86A2*, *CYP89A2* and *LAC2* genes was also suppressed, proving the link of *HvWIN1* in regulating these genes in cuticle biosynthesis as a defense response.

## Highlight

WAX INDUCER1 (HvWIN1) transcription factor regulates cutin biosynthetic genes to reinforce cuticle to resist Fusarium head blight. *CYP86A2*, *CYP89A2* and *LACS2* are potential downstream targets of HvWIN1.

## Introduction

About two-thirds of the global barley crop is used as animal feed, while the remaining one-third is used for malting, brewing and distillation (Newton *et al.*, 2011). Barley is not only critical for food and feed, but also has unparalleled impact on the social and economic development of several countries (McMullen *et al.*, 2012). However, the crop is severely affected by the devastating disease Fusarium head blight (FHB) caused by a fungus, *Fusarium graminearum* (Trail, 2009). Spores germinate and the hyphae enter through the space between the lemma and palea. Developing kernels are infected through the epicarp, which destroys layers of the seed coat and finally starch and protein in the endosperm (Jansen *et al.*, 2005). FHB management requires implementation of a variety of cultural and management practices. However, plant resistance through genetic improvement is considered to be the most efficient, economic and ecofriendly approach to reduce disease intensity (Bai and Shaner, 2004).

Five different types of resistance have been documented in the Triticaceae, three of which are often used in breeding wheat and barley (Mesterhazy, 1995). Since barley has high level of rachis resistance (type II), most research has focused on spikelet resistance (type I) and resistance to DON accumulation in grains (type III) (Zhu *et al.*, 1999). Resistance in plants against pathogen stress has been defined as the degree of susceptibility, ranging from high susceptibility (low resistance) to hypersensitive response (high resistance). The resistance is controlled by hierarchies of *R* genes, including genes that regulate downstream genes, which biosynthesize resistance-related (RR) metabolites and proteins (Kushalappa *et al.* 2016). The RR metabolites and proteins can be constitutive (RRC) or induced (RRI) (Kushalappa and Gunnaiah, 2013). Semi-targeted metabolomics have identified hundreds of metabolites involved in FHB resistance, and also the deposition of hydroxycinnamic acid amides (HCAA) and flavonoids that thicken cell walls to contain the pathogen to initial infection (Gunnaiah *et al.*, 2012). Several metabolic pathways have been shown to be upregulated in barley against *F. graminearum* infection (Bollina *et al.*, 2010; Kumaraswamy *et al.*, 2011*a*, *b*). Further, gene expression and silencing studies have been used to confirm the upregulation of genes involved in the HCCA biosynthetic pathway (Yogendra *et al.*, 2015*a*).

In our previous studies on FHB disease resistance in both barley and wheat, free fatty acids (FFAs) showed a higher fold change (FC) in resistant than in susceptible (Kumarswami *et al.*, 2011*a*, *b*; Gunnaiah *et al.*, 2012). The fatty acid metabolic pathway plays a significant role in plant defense against pathogen attack. For example, FFAs were implicated in their passive roles in plant defense by serving as biosynthetic precursors for the phytohormone jasmonic acid and cuticular components (Kachroo and Kachroo, 2009), which not only act as physical barriers but also as chemical antagonists against the invading pathogen. The cuticle is mainly made up of cutin monomers and oligomers, proposedly consisting of C16 and C18 fatty acids (Nawrath, 2002; Domínguez et al, 2015). Hydroxy and epoxy-hydroxy fatty acids of C16 and C18 fatty acids are interesterified to form cutin biopolyester. The cutin monomers also can form stable nanoparticles called cutinosomes which enable them to bind to each other to form layers (Domínguez *et al.*, 2015). More than 190 candidate genes involved in the cuticle biosynthetic components have been isolated and characterized, mostly in the model plant Arabidopsis, including *cytochrome P450* (*CYP*s), *glycerol-3-phosphate: acyl-CoA acyltransferase* (*GPAT*), *long chain acyl-CoA synthetases* (*LACS*) and members of *ATP binding cassette* (ABC) transporters (Xiao *et al.*, 2004; Yang *et al.*, 2014). The accumulated biochemical, physiological and genetic evidence of the cuticle in plant protection against biotic and abiotic stresses has stimulated search for gene networks underlying such protective responses (Borisjuk *et al.*, 2014). Cuticle biosynthetic genes are regulated by transcription factors. A transcription factor, of the ethylene response factor (ERF) family, WAX INDUCER1/SHINE1 (WIN1/SHN1), has recently been shown to induce the production of epidermal waxes when overexpressed in Arabidopsis plants. Analysis of *35S:WIN1* overexpressors suggested the role of *WIN1* in wax accumulation through direct or indirect regulation of metabolic pathway genes (Aharoni *et al.*, 2004; Broun *et al.*, 2004).

The aim of our study was to explore the biochemical and molecular basis of resistance in barley to FHB. Two barley genotypes with contrasting levels of resistance – a resistant (CI9831) and a susceptible (H106-371) – were mock- and pathogen-inoculated, and metabolites were profiled using liquid chromatography and high resolution mass spectrometry (LC-HRMS). The RR metabolites with high-fold change, especially the FFAs, were selected as candidate metabolites, mapped in metabolic pathways to identify the candidate genes, including a TF WAX INDUCER1 gene (designated herein as *HvWIN1*), which regulated them, based on genomic databases. The expression of these genes was confirmed based on quantitative real-time polymerase chain reaction (qPCR) studies. The RR genes in barley against FHB discovered here, based on metabolo-genomics, demonstrated the role of FFAs in cuticle biosynthesis in barley to resist *F. graminearum*. When the *HvWIN1* gene was silenced, based on virus-induced gene silencing (VIGS) in the resistant genotype, not only disease severity but also fungal biomass increased, confirming a shift from resistant to susceptible phenotype. Furthermore, it was associated with a decrease in abundance of several RR metabolites, whose biosynthetic genes were regulated by this gene, confirming its role in FHB resistance in barley. The potential application of this gene in breeding is discussed.

## Materials and methods

### Plant production

Two row barley genotypes varying in resistance to FHB, CI9831 (R, resistant) and H106-371 (S, susceptible), were selected based on field and greenhouse studies (Choo *et al.*, 2004; Kumar *et al.*, 2015). Five seeds of each genotype sown in pots filled with 50:50 pasteurized soil and 155 AgroMixR21 AF (Fafard, Quebec), thinned to three individuals following germination, were maintained in a greenhouse at 23±2 °C, 70±10% relative humidity and with a photoperiod of 16h throughout the growing period (Chamarthi *et al.*, 2014). Fertilizer solution (250ml), containing 0.3% of PlantProd (20:20:20 NPK) and 0.03% of trace elements was applied every 15 d to each pot.

### Fungal spore production and inoculation

*F. graminearum* was grown in potato dextrose agar (PDA) plates and incubated at 26 °C for 4 d. For spore production, pathogen was further sub-cultured in Rye B agar plates and kept inverted for another 4 d by exposing the plates to 8h dark and 16h of near-UV light. Macroconidia were harvested from 7-day-old cultures, spore concentration was determined using a hemocytometer (American Scientific Products, USA) and finally adjusted to 1×10^5^ macroconidia ml^−1^ (Bollina *et al.*, 2010).

Three alternate pairs of barley spikelets at 50% anthesis stage, were point inoculated with 10 μl of either macroconidial suspension in water or mock-solution using a syringe (GASTIGHT 1750 DAD, Reno, USA). After inoculation, plants were covered with polyethylene bags sprayed with water to maintain enough moisture inside. Bags were removed 48h post inoculation (hpi).

### Experimental design and metabolite analysis using LC-HRMS

The experiment was conducted as a completely randomized block design, with the two genotypes with contrasting levels of resistance to FHB (CI9831 and H106-371), two inoculations (pathogen and mock), with five replicates over time at 3 d intervals. Each replicate, or the experimental unit, consisted of 48 pairs of spikelets, collected from eight spikes in three plants; from each spike three alternate pairs of spikelets inoculated with either macroconidial suspension or mock-solution and three alternate pairs of uninoculated spikelets were harvested. The samples were frozen immediately in liquid nitrogen and stored at −80 °C until use. Metabolite analysis was performed as described earlier (Gunnaiah *et al.*, 2012; Yogendra *et al.*, 2014, 2015*b*). In brief, 100mg of spikelets were ground in liquid nitrogen, and metabolites were extracted in 60% ice-cold methanol, to which 200 pg of genistin was added as internal control (Chamarthi *et al.*, 2014). The extract was sonicated for 15min at 25 °C using a waterbath sonicator (Mesonix, USA) and 5 µl of resulting clear supernatant was used for metabolite analysis. These were analyzed in negative ionization mode using the LC-HRMS system (LC-ESI-LTQ Orbitrap, Thermo Fisher, USA), using a 5cm kinetex column.

### Identification of resistance-related (RR) metabolites

The data on intensity of peaks of monoisotopic masses (m/z = mass/charge ratio, subtracted with a proton mass because of negative ionization) were subjected to pairwise Student’s *t*-test analysis (SAS v 9.3). The treatment combinations tested were RM vs SM, RP vs RM, and SP vs SM, where R=resistant, S=susceptible, M=mock, and P=pathogen-inoculated. The peaks significant at *P*<0.05 (Kushalappa and Gunnaiah, 2013), relative fold change of ≥2 and false discovery rate threshold of 0.05 (Vinaixa *et al.*, 2012) were retained. False discovery rate of peaks depends mainly on the signal/noise (S/N) ratio; the lower the ratio, the higher are the false discovery rates. Therefore, the S/N ratio was kept high to avoid any false discovery. However, this was only used as initial selection criteria, to include as many metabolites as possible. The significant abundances of 756 metabolites, present in all five replicates, were subjected to canonical discriminant analysis using the CANDISC procedure (SAS v 9.3) to classify the observations. The data dimension was reduced by a nonsupervised principal component analysis, and the principal components were subjected to supervised discriminant analysis to classify the observations to treatments. The canonical discrimination analysis (CAN) scores were used to develop a scatterplot which discriminated the treatments and identified resistance functions (Hamzehzarghani *et al.*, 2005). The loadings of metabolites to CAN vectors were used to interpret the results.

The metabolites with significantly higher abundances in R than in S, based on a *t*-test, were considered as RR metabolites. These were further grouped into RR constitutive (RRC=RM>SM) and RR-induced (RRI=(RP>RM)>(SP>SM)) metabolites. For these RR metabolites, the fold change (FC) in abundance, relative to susceptible (R/S), was calculated. The RR metabolites were putatively identified based on two criteria: (i) the accurate mass match [accurate mass error (AME<5ppm)] with metabolites reported in different databases: METLIN, KNApSAcK, Plant Metabolic Network (PMN), LIPIDMAPS and KEGG; (ii) the fragmentation pattern match with those in databases or *in silico* verification (Gunnaiah *et al.*, 2012). The metabolites were mapped on metabolic pathways using a pathway tool omics viewer searched against Arabidopsis and *Hordeum vulgare* metabolites (Bollina *et al.*, 2010).

### Prediction of genes involved in cuticle biosynthesis

Since the cuticle biosynthetic metabolites were highly accumulated in the resistant genotype, the reported gene sequences involved in cuticle biosynthesis in Arabidopsis and other plants were BLAST-searched using the assembly_WGSMorex IPK Barley BLAST server (http://www.public.iastate.edu/~imagefpc/IBSC%20Webpage/IBSC%20Template-home.html). The contigs were downloaded and genes were predicted using the SoftBerry FGENESH program (http://linux1.softberry.com/berry.phtml?topic=fgenesh&group=programs&subgroup=gfind).

### RNA extraction, cDNA synthesis and quantitative real-time PCR analysis

Total ribonucleic acid (RNA) was extracted from the ground spikelets using the RNeasy Plant mini kit (Qiagen, Germany) and treated with DNase I as per the supplier’s recommendations. Purified RNA (500ng from each sample) was reverse transcribed using the iScript cDNA synthesis kit (BioRad, ON, Canada). Two microliters of 40×-diluted cDNA was used in a qPCR reaction using the iQ SYBR Green Supermix (BioRad) in an iQ CFX384^TM^ Real-Time System (Bio-Rad, Mississauga, ON, Canada). Primer sequences and PCR conditions used for actin and other genes are given in Supplementary Table S1 at *JXB* online.

### Cloning, sequencing and bioinformatics analysis of the *HvWIN1* transcription factor

Amplification of the full length sequence of *HvWIN1* from barley cultivars CI9831 and H106-371 was carried out in a 25 μl volume using both genomic DNA and cDNA as template and gene specific forward (5ʹ-ATGGCGGTCGAGTTCGGGAATTTTG-3ʹ) and reverse primer pair (5ʹ-CTATGACGAGGCTGCCGTTCTGAT-3ʹ) designed from start and stop codon, respectively. Gene amplification was conducted using a thermal cycler (Bio-Rad, Mississauga, ON, Canada) with the following steps: heat denaturation at 94 °C for 3min, followed by 35 cycles of 94 °C for 30s, annealing at 58 °C for 40s, extension at 72 °C for 2min and final extension at 72 °C for 7min. The amplified PCR product was cloned into the pGEM^®^-T Easy vector (Promega, USA) and sequenced using the ABI Automated DNA Sequencer. DNA sequences were translated to amino acid sequences using the ExPASy Translate Tool (http://web.expasy.org/translate/). Multiple sequence alignments of nucleotide and amino acids were done using MultAlin (http://multalin.toulouse.inra.fr/multalin/) to identify polymorphisms in the sequences of resistant and susceptible genotypes. To check the polymorphic status, *HvWIN1* was also cloned and sequenced from three other cultivars, namely Zhedar-2, AC-Metcalfe and CDC Copeland. All the sequences were submitted to the NCBI database. The sequences were aligned with barley reference sequence using multalin software (http://multalin.toulouse.inra.fr/multalin/). A phylogenetic tree was built using the à la carte function on the Phylogeny.fr server (http://www.phylogeny.fr/).

### Construction of BSMV-derived vector, *in vitro* transcription of viral RNAs and plant inoculation for virus-induced gene silencing (VIGS) of *HvWIN1* TF

For transient gene silencing, the target 250bp *HvWIN1* (GenBank ID: KT946819) fragment was amplified using forward (5ʹ-TGGGTCTCCGAGATCAGAC-3ʹ) and reverse primers (5ʹ-GAAGCTTGGCACTGAGGAC-3ʹ). This region was chosen from the N-terminus region of the gene, which on BLAST N and BLAST X analysis on the NCBI server did not show homology with any other barley genes. Also, BLAST analysis on the IPK BLAST server (http://webblast.ipk-gatersleben.de/barley) revealed very low homology scores with other genes (Supplementary Table S2). The fragment was checked for genome-wide off-target siRNAs using the SGN VIGS Tool (http://vigs.solgenomics.net/) with 250bp fragment size and n-mer of 21bp using *Brachypodium distachyon* v3.1 as query database. Result of this analysis showed homology with the Bradi3g07450.1 gene, which on analysis was found to be the ethylene-responsive transcription factor *WIN1*-like gene. The fragment was cloned in pGEM^®^ T-Easy vector (Promega, USA) followed by digestion of plasmid DNA with *NotI* enzyme, thereby producing *Not1* ends. The cDNA fragment was subsequently ligated to the pSL038-1 vector, a plasmid encoding a modified BSMV modified γ genome segment with a cloning site downstream of the γb gene (Cakir and Scofield, 2008). The pSL038-1 vector carrying either the *phytoene desaturase* (*PDS*) gene or without any plant gene served as positive control and negative controls, respectively. The plasmids pα46 (BSMV α) and pγSL038-1 were linearized with the *Mlu1* restriction enzyme, whereas pβ42sp1 (BSMV β) was linearized by using the *Spe1* enzyme. All linearized plasmids were converted to capped *in vitro* transcripts using mMESSAGE mMACHINE® T7 *in vitro* transcription kit (Ambion, Inc., Austin, TX, USA) following the manufacturer’s protocol. A 20 μl reaction contained 10 μl of 2× dNTPs, 1 μg of linearized plasmid, 2 μl of 2× buffer, 2 μl of enzyme mix, and water to a final volume of 20 μl. The reaction setup was scaled up as per the requirements of the experiment. The reaction mix was incubated at 37 °C for 2h and *in vitro* transcription was confirmed by running 1 μl of transcript with 9 μl of RNase-free water on 1% agarose gel.

Flag leaf and spikelets of the resistant genotype CI9831 were rub-inoculated, at growth stage 50–55 (Zadoks *et al.*, 1974), with all the three *in vitro* transcript reactions (α, β and γ BSMV) in a 1:1:1 ratio (1 µl of each was used) along with 22.5 μl inoculation buffer (IB) that facilitated viral infection as it contained abrasive material (Scofield *et al.*, 2005; Ma *et al.*, 2012). The experimental units consisted of two plants, with a total of six spikes inoculated separately with test treatment (BSMV+*HvWIN1*) and negative control (BSMV:00), with five biological replicates over time. Barley spikelets were also rub-inoculated with positive controls (BSMV+PDS). Twelve days after silencing, three alternate pairs of spikelets in three spikes were inoculated with 10 µl of either mock or macroconidial suspension (1×10^5^ macroconidia ml^‒1^) for both negative control and test treatment. Plants were covered with plastic bags to maintain high humidity. Spikelet samples for metabolite and gene expression studies were collected 3 dpi (days post-inoculation) whereas, for fungal biomass quantification, these were collected 6 dpi.

### Fungal biomass quantification

Genomic DNA was isolated from barley spikelets infected with *F. graminearum* (6 dpi) using a DNeasy Plant Mini Kit (Qiagen, Canada). Relative biomass of *F. graminearum* in the infected samples was quantified by quantitative real-time PCR (qPCR) as described by Kumar *et al.* (2015). PCR was performed in iQ CFX384^TM^ Real-Time System (Bio-Rad, Mississauga, ON, Canada) using primers for *Tri6* as target gene and *Actin* as a housekeeping gene and an equal quantity of genomic DNA (20ng) of each sample. The relative fungal biomass was estimated by normalizing the Ct values for *Tri6* to that of barley *Actin* gene and calculating the relative gene copy number of *Tri6* using the 2^˗ΔΔC^_T_ method (Livak and Schmittgen, 2001).

## Results

### Canonical discriminant analysis discriminated resistance and pathogenesis functions in plant-pathogen interaction

Canonical discriminant analysis was used to classify the observations, abundances of 1864 peaks consistent in all the five replicates and to explain resistance or pathogenicity functions. The five replicates of each variable were clustered in one group, meaning that the experimental error was minimal. The CAN1 vector explained 92% variance, discriminating the resistant and susceptible genotypes. The CAN2 vector explained only 4% variance, discriminating the inoculations, explaining the pathogenesis, meaning the CAN vectors mainly explained the resistance function (Supplementary Fig. S1).

### Semi-targeted metabolomics analysis revealed FFAs as high-fold resistance-related induced metabolites

**Fig. 1.**
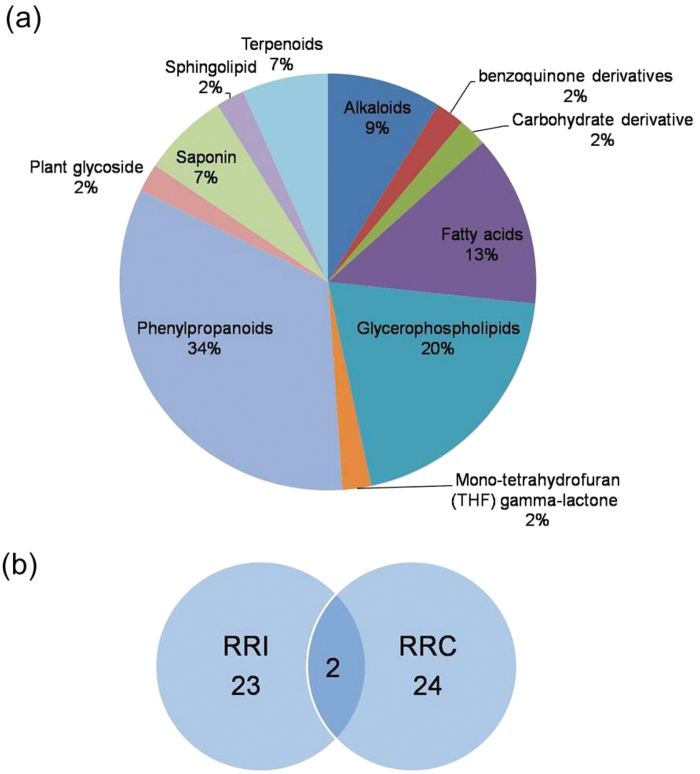
Analysis of resistance-related (RR) metabolites. (A) Classification of RR metabolites according to chemical groups. (B) Venn diagram showing number of RR metabolites (>2-fold) identified in the spikelets of barley cultivar CI9831, where spikelets were inoculated with water or *F. graminearum* spores. RRI, resistance-related induced metabolites; RRC, resistance-related constitutive metabolites. A colour version of this figure is available at *JXB* online.

Metabolites were profiled in the spikelets of resistant and susceptible genotypes at 3 dpi, inoculated with *F. graminearum* or with water and analyzed based on LC-HRMS. A total of 1864 monoisotopic peaks were detected and the significant ones were categorized into RRC and RRI metabolites. Data was reduced by considering only those metabolites that had abundances FC>2. Out of these 47 high FC metabolites, 24 were identified as RRC metabolites and 21 as RRI metabolites (Table 1; Fig. 1A, B; Supplementary Table S3; Supplementary Fig. S2). The majority of the high FC RRI metabolites belonged to the fatty acid pathway. Among these, five FFAs were induced with high-fold change: linoleate (FC=39.00), palmitic acid (FC=6.56), sebacic acid (FC=2.79), arachidonoyl m-Nitroaniline (FC=2.60), auricolic acid (FC=2.05).

**Table 1.** Resistance-related metabolites detected in the spikelets of barley genotypes inoculated with water or spores of *F. graminearum.* RRI, resistance-related induced metabolites; RRC, resistance-related constitutive metabolites.

**Observed mass** **(Da)**	**Exact mass** **(Da)**	**Name**	**Relative fold change (FC**)
**Alkaloids**			
326.2005	326.1994	Ajmaline	2.44 RRI**, 2.03 RRC**
340.2158	340.2150	(+)-Sandwicolidine	2.10 RRI*
449.2769	449.2777	Condelphine	23.00 RRI***
596.3533	596.3515	C-Curarine	3.26 RRI*, 2.03 RRC**
**Fatty acids**			
202.1210	202.1205	Sebacic acid	2.79 RRI*
256.2405	256.2402	Palmitic acid	6.56 RRI**
280.2404	280.2402	Linoleate	39.00 RRI***
324.2674	324.2664	Auricolic acid	2.05 RRI*
390.1893	390.1890	(-)-11-hydroxy-9,10-dihydrojasmonic acid 11-beta-D-glucoside	2.75 RRC**
424.2723	424.2726	Arachidonoyl m-Nitroaniline	2.60 RRI**
**Glycerophospholipids**			
410.2435	410.2433	1-Palmitoylglycerol 3-phosphate	2.43 RRI*
572.2961	572.2962	PI (16:0/0:0) or 1-hexadecanoyl-sn-glycero-3-phospho-(1’-myo-inositol)	4.21 RRI*
610.3843	610.3846	PG (12:0/12:0) or 1,2-didodecanoyl-sn-glycero-3-phospho- (1’-sn-glycerol)	2.73 RRC**
740.4631	740.4628	PG (14:1(9Z)/20:4(5Z,8Z,11Z,14Z))	5.44 RRC**
754.4414		PC (5:0/30:11)	3.32 RRC*
772.4521		PS (0:0/37:10)	2.67 RRC*
788.4469	788.4476	PI (13:0/18:4(6Z,9Z,12Z,15Z))	2.04 RRC**
790.4656	790.4632	PI (13:0/18:3(6Z,9Z,12Z))	2.07 RRC*
956.8783	956.8772	TG (19:0/20:1(11Z)/20:1(11Z))[iso3]	2.84 RRC*
**Sphingolipids**			
450.3218	450.3223	C17 sphingosine-1-phosphocholine	2.69 RRI*
**Phenylpropanoids**			
**Phenylpropanoid (phenolics**)			
476.2177	476.2198	Fuscaxanthone A	2.08 RRC*
**Phenylpropanoid (hydroxycinnamic acid amides**)			
360.1057	360.1056	Syringic acid beta-glucopyranosyl ester	3.50 RRC*
**Phenylpropanoids (flavonoids**)			
270.0531	270.0528	Apigenin	2.46 RRC**
338.1164	338.1154	(-)-Glyceollin I	5.50 RRC*
372.1211	372.1209	Sinensetin	2.20 RRI*
432.1061	432.1056	Pelargonidin 3-O-glucoside	2.91 RRC*
506.1062	506.1060	Quercetin 7-(6’’-acetylglucoside)	2.96 RRC**
578.1642	578.1636	Rhoifolin	12.12 RRC**
596.1728	596.1741	Isobutrin	2.05 RRC*
608.2114	608.2105	Matteucinol 7-O-beta-D-apiofuranosyl(1->6)-beta-D-glucopyranoside	2.22 RRC*
740.2162	740.2164	Robinin (kaempferol-3-O-robinoside-7-O-rhamnoside)	4.84 RRC**
386.2668	386.2668	6-Deoxyerythronolide B	2.51 RRI*
**Phenylpropanoids (lignans**)			
418.1631	418.1628	Lirioresinol A, (+)-Syringaresinol	2.16 RRC*
596.2439	596.2410	Magnolignan G	2.29 RRI*
748.2949	748.2942	Alangisesquin C	4.53 RRC**
**Saponin**			
740.4347	740.4347	Timosaponin A-III	2.08 RRI*
868.5170	868.5184	Nephelioside I	2.04 RRI*
870.4979	870.4976	Racemoside C	3.12 RRI*
**Terpenoids**			
446.2294	446.2305	Glycinoeclepin A	7.73 RRI**
596.3964	596.3865	Astaxanthin	2.14 RRC*
732.4579	732.4601	3-(E)-Coumaroylbetulin-28-yl-ethyl (2R)-2-hydroxysuccinate	2.68 RRC*

Detailed compound identification is presented in Supplementary Table S3. Fold change calculation was based on relative intensity of metabolites: RRC=RM/SM and RRI=(RP/RM)/(SP/SM). Significance (*t*-test): *, *P*<0.05; **, *P*<0.01; ***, *P*<0.001.

### Transcript expression of barley genes involved in cuticle biosynthesis

Based on literature, some of the important genes involved in fatty acid and cuticle biosynthesis were selected for transcript expression analysis. These genes were *KAS2*, *CYP86A2, CYP89A2, LACS2, GPAT6* and *CER5*; which were considered to be involved in the production of ω-hydroxy fatty acid components that formed the cutin polymer (Smith *et al.*, 2000; Kannangara *et al.*, 2007). Detailed information on the predicted gene sequences in the barley genome – including gene size, chromosomal localization and expression primers used for qPCR – is given in Supplementary Table S4 and Supplementary Table S1, respectively.

**Fig. 2.**
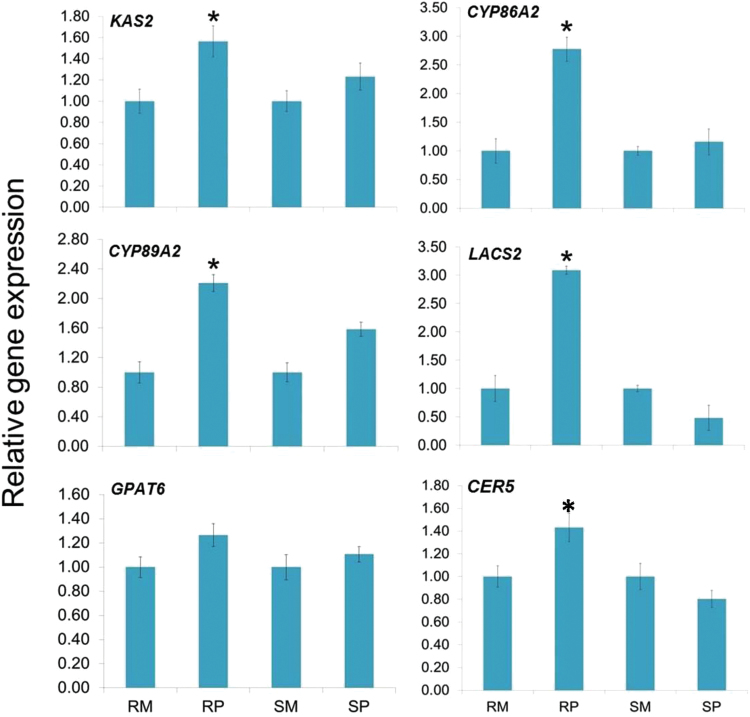
Relative transcript expression of *RR* genes from spikelet samples of resistant and susceptible genotypes following *F. graminearum* and mock inoculation at 72 hpi based on qPCR in comparison to reference gene *Actin*. CI9831 and H106-371 are resistant and susceptible genotypes, respectively. Error bars represent ±SE. Significant differences in relative expression levels using Student’s *t*-test: *, *P*<0.05. A colour version of this figure is available at *JXB* online.

β-ketoacyl-(acyl carrier protein) synthase 2 (*KAS2*) is a key gene involved in the biosynthesis of FFAs, which catalyzes the biosynthesis of malonyl and stearate (utilizing palmitoyl-ACP or myristoyl-ACP for condensation with malonyl-ACP and controlling the final ratio of C16/C18 products). *KAS2* showed enhanced gene expression in pathogen-inoculated compared to mock-inoculated genotypes (Fig. 2). The expression of *CYP86A2/CYP89A2* that catalyzes the oxidation of fatty acids and diverts metabolic flux toward cutin biosynthesis, was significantly (*P*<0.05) increased in the resistant genotype, following pathogen inoculation, compared to mock inoculation. No significant difference in gene expression was observed between mock and pathogen-susceptible genotype (Fig. 2) for *CYP86A2*. *LACS2* is involved in the conversion of ω-hydroxy fatty acids to their CoA thioesters. Whereas the expression of *LACS2* was significantly (*P*<0.05) increased in the pathogen-inoculated resistant (CI9831) genotype, as compared to the susceptible genotype H106-371, in which it was actually decreased. In parallel, the *GPAT6* that catalyzes the conversion of FFAs to ω-OH-acylglycerol, was also higher in both CI9831 and H106-371, inoculated with pathogen, but was not significant. Similarly, expression of *CER5*, an ABC transporter, considered to be involved in cuticular lipid export was also higher in pathogen-inoculated compared to mock-inoculated resistant genotype CI9831. Conversely, the expression of *CER5* was reduced in pathogen-inoculated susceptible H106-371 genotype, as compared to mock-treated samples (Fig. 2).

### Differential transcript expression of *HvWIN1*: a potential transcriptional regulator of cuticle biosynthetic genes in barley

**Fig. 3.**
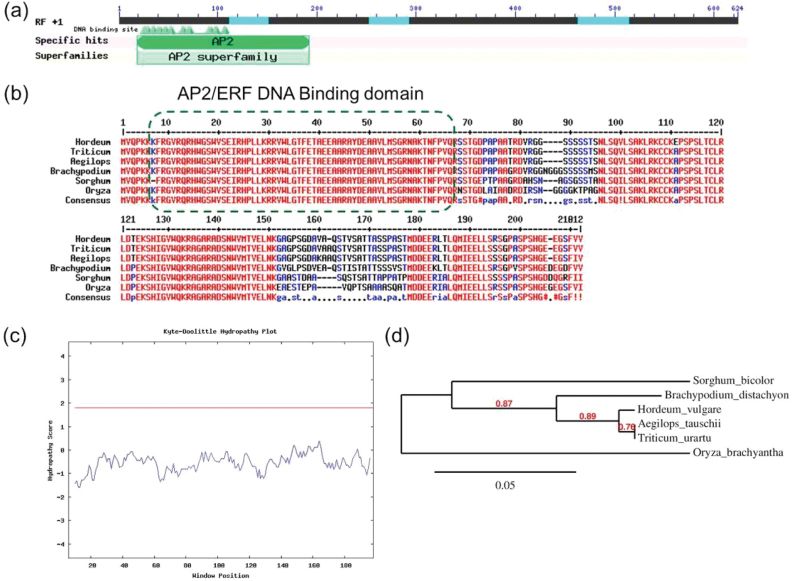
*In silico* analysis of *HvWIN1*. (A) Catalytic domain prediction by NCBI Conserved Domain Database Search. (B) Multiple sequence alignment of *HvWIN1* from closely related plant species showing AP2/ERF DNA binding domain. (C) Hydropathy plot analysis at a window size of 19. Peaks with scores greater than 1.8 (horizontal line) indicate possible transmembrane regions. (D) Phylogenetic relationships of *HvWIN1* with other plant *WIN1* sequences retrieved from the NCBI database. The phylogenetic tree was built using the ‘à la carte’ function on the Phylogeny.fr server. A colour version of this figure is available at *JXB* online.

**Fig. 4.**
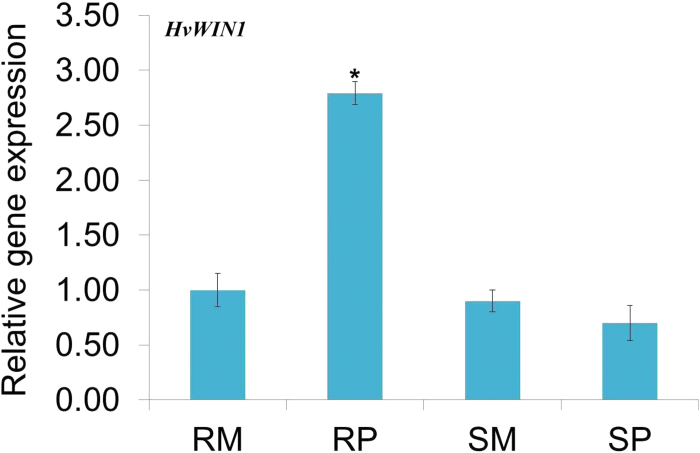
Relative transcript expression of *HvWIN1* TF. The relative transcript expression was measured in the mock- and pathogen-treated resistant and susceptible genotypes compared with the mock-inoculated resistant genotype at 72 hpi. Samples: RM, resistant mock-treated; RP, resistant pathogen-treated; SM, susceptible mock-treated; SP, susceptible pathogen-treated. Significant differences in expression levels as compared in RP compared with SP using student’s *t*-test: *, *P*<0.05. A colour version of this figure is available at *JXB* online.

Previous studies in Arabidopsis have shown *WIN1* as a transcriptional regulator of genes involved in cutin biosynthesis (Aharoni *et al.*, 2004; Broun *et al.*, 2004; Kannangara *et al.*, 2007). Further, we studied the potential interacting partners of WIN1 using WIN1/SHN1 as query and Arabidopsis as search organism in STICH 4.0 software (http://stitch.embl.de). STITCH is a resource used to explore known and predicted interactions of chemicals and proteins. Chemicals are linked to other chemicals and proteins by evidence derived from experiments, databases and the literature (Kuhn *et al.*, 2014). The resulting network clearly showed direct interaction of WIN1 TF with downstream targets like *LACS2*, *GPAT4*, *CYP86A4* and *CER5* (Supplementary Fig. S3) and also with palmitate, with high FC observed in our study. Therefore, *WIN1* was considered as a potential TF to explore further. WIN/SHINE TF, known to be involved in cutin biosynthesis from Arabidopsis (GenBank accession no. NM_101405), was BLAST searched in the IPK Barley BLAST server (http://webblast.ipk-gatersleben.de/barley/) in the assembly_WGSMorex database. BLAST hit showed maximum homology to morex_contig_1564026. *HvWIN1* was predicted in this contig using FGENESH software (http://www.softberry.com). Primers were designed for full length amplification of *HvWIN1* from barley genotypes under study. Sequence analysis showed *HvWIN1* to have a coding sequence of 621bp that encoded 206 amino acids having a molecular mass of 22.27kDa and a pI of 9.47. There was no difference in the coding sequence of *HvWIN1* between the resistant (CI9831) and susceptible genotype (H106-371), however, the susceptible allele revealed an additional intron between nucleotides 83 and 189 (Supplementary Fig. S4). To check the polymorphic status, *HvWIN1* was also cloned from other barley FHB susceptible genotypes (Supplementary Table S5). The presence of an additional intron was a common feature for all tested susceptible genotypes (Supplementary Fig. S4). Bioinformatics analysis revealed *HvWIN1* to have an AP2 domain (Fig. 3A), which is a DNA-binding domain found in transcription regulators in plants such as APETALA2 and EREBP (ethylene-responsive element binding protein). In EREBPs, the domain specifically binds to the 11bp GCC box of the ethylene response element (ERE), a promotor element essential for ethylene responsiveness. Multiple sequence analysis of *HvWIN1* from other plant species suggested AP2 domain to be highly conserved (Fig. 3B). Hydropathy plot analysis suggested *HvWIN1* to be a cytosolic protein with no transmembrane domains (Fig. 3C). Phylogenetic analysis showed a close relatedness of barley *HvWIN1* to those of *Aegilops tauschii* and *Triticum urartu* (Fig. 3D). Accordingly, the gene that we cloned from barley was designated here as *HvWIN1*. To study the role of *HvWIN1* in FHB disease resistance, we carried out transcript expression analysis using qPCR. *HvWIN1* was significantly (*P*<0.05) upregulated, following pathogen invasion, compared to mock treatment in the resistant genotype (CI9831) (Fig. 4). No significant difference was observed in the susceptible genotype (H106-371) inoculated with the pathogen as compared to mock inoculation (Fig. 4), suggesting its potential role in FHB resistance in the resistant genotype.

### Silencing of *HvWIN1* gene in the resistant genotype rendered it susceptibility to FHB

**Fig. 5.**
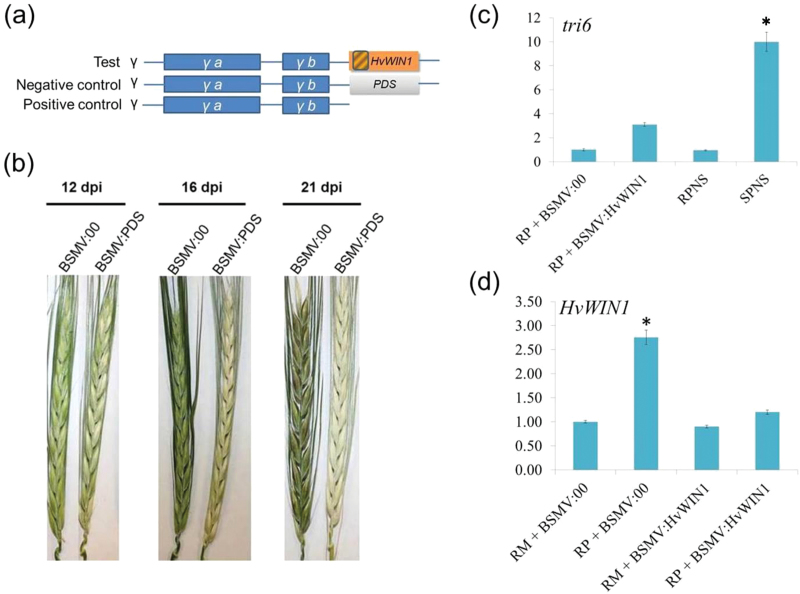
Virus-induced gene silencing of *HvWIN1*. (A) Schematic representation of BSMV-based virus-induced gene silencing (VIGS) vectors. The *HvWIN1* cDNA fragment was cloned to pSL038-1 vector (modified BSMV vector) downstream of the γb gene (BSMV:*HvWIN1*). The vector carrying either the *phytoene desaturase* (*PDS*) gene (BSMV:*PDS*) or without any plant gene (BSMV:00) served as positive control and negative control, respectively. The region in the *HvWIN1* with gradient fill depicts the part of the gene used for silencing. (B) Phenotypes of the spikes from barley cultivar CI9831 rub-inoculated with either BSMV:*PDS* or BSMV:00 constructs. Spikes were rub-inoculated with either BSMV:00 (negative control) or BSMV:*PDS* (positive control) constructs at flag leaf stage and photographed at different time intervals. dpi, days post rub-inoculation. (C) Relative fungal biomass calculated as relative gene copy number of *tri6* at 6 dpi. (D) Relative transcript expression measured at 72 hpi. RM, mock-treated resistant samples; RP, pathogen-treated resistant samples; BSMV:00, barley plants rub-inoculated with negative control; BSMV:*HvWIN1*, barley plants rub-inoculated with BSMV vector containing *HvWIN1*. Significant differences in expression levels in RP+BSMV:00 compared with RP+BSMV:*HvWIN1* using student’s *t*-test: *, *P*<0.05. A colour version of this figure is available at *JXB* online.

To prove the resistance function of *HvWIN1* in barley against FHB, the *HvWIN1* gene in the resistant plant was knocked down by VIGS. The schematic representation of the VIGS experiment is shown in Fig. 5A. Plants expressing the pSL038-1 vector carrying the *PDS* gene (involved in the carotenoid metabolic pathway) exhibited photobleaching after 12 d of rub inoculation, which served as positive controls confirming the success of the silencing protocol (Fig. 5B). Complete photobleaching was observed at and after 16 dpi (Fig. 5B). To assess the effect of *HvWIN1* in FHB resistance, the *F. graminearum* biomass was quantified in silenced and non-silenced resistant plants. Silencing of *HvWIN1* in CI9131 led to a significant increase (*P*<0.005) in copy number of the *Tri6* gene (3-fold), depicting the amount of fungal biomass, compared to non-silenced control plants (Fig. 5C). Transcript abundance analysis of *HvWIN1* using qPCR showed substantial reduction in copy number due to knockdown of the gene (Fig. 5D). These results suggested that the observed phenotypic difference in FHB resistance was due to *HvWIN1* silencing.

### Silencing of *HvWIN1* reduced fatty acid RR metabolites and led to reduced transcript abundance of genes involved in cutin biosynthesis

**Fig. 6.**
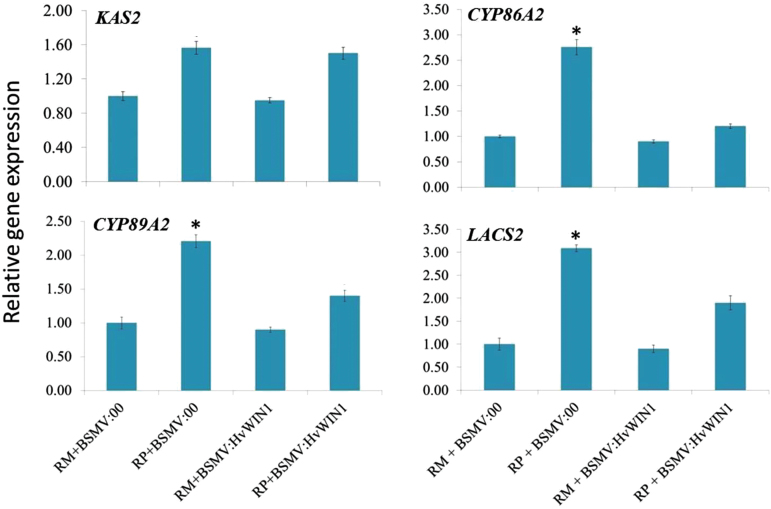
Effect of *HvWIN1* silencing on relative transcript expression of downstream target genes. The relative transcript expression at 72 hpi was measured by comparing the expression with mock-inoculated resistant genotype. RM, mock-treated resistant genotype samples; RP, pathogen-treated resistant genotype samples; BSMV:00, barley plants rub-inoculated with negative control; BSMV:*HvWIN1*, barley plants rub-inoculated with BSMV vector containing *HvWIN1*. Significant differences in expression levels in RP+BSMV:00 compared with RP+BSMV:*HvWIN1* using student’s *t*-test: *, *P*<0.05. A colour version of this figure is available at *JXB* online.

As discussed in the previous section, *HvWIN1*-silenced barley spikes exhibited lower levels of resistance to FHB; accordingly, the plausible biochemical and molecular mechanisms of involvement of *HvWIN1* to resist FHB were further explored. Semi-targeted metabolomic analysis was carried out to confirm the effect of *HvWIN1* silencing on secondary metabolite production. The FFA RRI metabolites palmitic acid (FC=6.56–1.3) and linoleate (FC=39.00–3.09) were significantly reduced (*P*<0.05) after *HvWIN1* silencing (Table 2), suggesting the potential involvement of *HvWIN1* in the FFA biosynthetic pathway. Other FFAs such as sebacic acid (FC=1.08), arachidonoyl m-Nitroaniline (FC=1.20) and auricolic acid (FC=1.10) were also detected in less abundance (Table 2). To further study the downstream targets of *HvWIN1*, we carried out promoter analysis of genes for which promoter sequences were available. One thousand bases above the transcription start site were considered for analysis using the bioinformatics tool PlantCare (http://bioinformatics.psb.ugent.be/webtools/plantcare/html/). Database sequences were available for *CYP86A2, CYP89A2, LACS2* and *GPAT6*. However, for *CYP89A2* only 400bp were available in the contig. Promoter analysis of these genes, however, failed to reveal any GCC box necessary for *HvWIN1* to bind to these promoters (Supplementary Table S6). Since *KAS2*, *CYP86A2* and *CYP89A2,* and *LACS2* had the highest expression upon pathogen inoculation, these were studied for gene expression after *HvWIN1* silencing. There was no significant difference in expression of *KAS2* upon *HvWIN1* silencing (Fig. 6). However, transcript abundance was significantly reduced for *CYP86A2* and *CYP89A2,* and *LACS2* upon pathogen treatment (Fig. 6), suggesting these as potential targets of *HvWIN1*.

**Table 2.** Effect of *HvWIN1* silencing on resistance-related fatty acid metabolites upon *F. graminearum* or mock-solution inoculation.

**Observed mass (Da**)	**Exact mass (Da**)	**Name**	**Fold changes before silencing**	**Fold changes after silencing**
280.2404	280.2402	Linoleate	39.00 RRI***	3.09*
256.2405	256.2402	Palmitic acid	6.56 RRI**	1.30*
202.1210	202.1205	Sebacic acid	2.79 RRI*	1.08*
424.2723	424.2726	Arachidonoyl m-Nitroaniline	2.60 RRI**	1.20*
324.2674	324.2664	Auricolic acid	2.05 RRI*	1.10*

Fold change calculation was based on relative intensity of metabolites: RRC=RM/SM and RRI=(RP/RM)/(SP/SM). Significance (*t*-test): *, *P*<0.05; **, *P*<0.01; ***, *P*<0.001.

## Discussion

Plants grown in the field have to face several environmental stresses. One of the mechanisms that protect the plants from biotic stresses is the formation of lipophilic barriers in the form of cutin or suberin (Yang *et al.*, 2012). Cutin and suberin are polymers of fatty acid derivatives linked by ester bonds, i.e. polyesters (Beisson *et al.*, 2012). These insoluble polymers, and associated waxes, function to control water loss, gas and ion fluxes, and also function as physical barriers to protect plants from pathogen invasion (Schreiber, 2010). These are mainly composed of ω-oxidized fatty acids (ω-OHFA), namely α,ω-dicarboxylic acids (DCAs), with a varying range of glycerol (Yang *et al.*, 2010). Cutin is mostly composed of C16 and C18 ω-hydroxy acids, polyhydroxy acids, epoxyacids and dicarboxylic acid (DCA) (Domínguez *et al.*, 2015). Although these are among the most abundant lipid monomers in plants, the genes involved in cutin biosynthesis are largely unknown.

*F. graminearum* enters through the space between lemma and palea, then infects cereal florets through natural openings, such as stomata, or by direct penetration (Bushnell, 2001). After penetrating the cuticle, the fungal hyphae grow subcuticularly in the tissues of glume, palea and lemma (Kang and Buchenauer, 2000; Jansen *et al.*, 2005). Fungal cutinases along with other cell wall degrading enzymes such as lipases are overexpressed during the infection process. Plants can defend against fungus by orchestrating a battery of genes to biosynthesize metabolites with either antimicrobial functions or by channelizing its resources to form structures that can confine the pathogen to the initial infection site. The epidermal cuticle is the first level of resistance the fungal pathogen has to overcome (Wanjiru *et al.*, 2002). A number of genes have been reported to be involved in biosynthesis of the cuticle in Arabidopsis (Yeats and Rose, 2013). However, in barley the specific metabolites or the genes involved in biosynthesis of the cuticle, especially its reinforcement to contain pathogens, have not been reported.

In the present study, an integrated metabolomics and functional genomics approach was used to explore mechanisms of resistance in barley against FHB, using two two-row barley genotypes, CI9831 and H106-371, with contrasting levels of resistance. A semi-comprehensive metabolomics analysis revealed a high-fold accumulation of FFAs, which were not only constitutively present but also induced following pathogen invasion (Table 1; Supplementary Table S3). The FFAs can be channelized to pathways such as the octadecanoid pathway for the biosynthesis of jasmonic acid and its derivatives, which further trigger several downstream defense-related target genes. However, in our study the FFAs such as palmitic acid and linoleic acid, along with glycerolipids such as 1-palmitoylglycerol 3-phosphate and PI (16:0/0:0) or 1-hexadecanoyl-sn-glycero-3-phospho-(1’-myo-inositol), produced high FC in abundance (Table 1), appeared to have been used as monomeric units to biosynthesize cutin (Domínguez *et al.*, 2015). These results tempted us to explore the possible mechanism of cutin biosynthesis as one of the major defense strategies in response to pathogen attack. Therefore, we conducted a stepwise approach in qPCR analysis for some of the genes involved in the biosynthesis of FFAs and further down to cutin biosynthesis. Genes known to be involved in cuticle biosynthesis from Arabidopsis were selected for BLAST analysis to identify those showing maximum homologies in the barley genome database.

*KAS2* is one of the important genes involved in FFA biosynthesis inside plastids (Beld *et al.*, 2015). Through analysis of mutants, some members of the *LACS* family, *cytochrome P450 oxidases* and *GPAT* have been shown to be required for cutin biosynthesis (Pollard *et al.*, 2008). *LACS* family members are required to activate FFAs to acyl-CoA for use by *GPATs* whereas members of the *P450* family, such as *CYP88* and *CYP77A*, were reported to be involved in ω-hydroxylation and midchain hydroxylation of FFAs (Pollard *et al.*, 2008; Grausem *et al.*, 2014). In barley, transcriptional silencing of *CYP96B22* based on VIGS led to a decrease in penetration resistance of barley plants to *Magnaporthe*, host and nonhost isolates (Delventhal *et al.*, 2014). GPAT catalyzes the transfer of an acyl group from acyl-CoA or acyl-ACP to the sn-1 position of the sn-glycerol-3 phosphate (G3P) (Murata and Tasaka, 1997). More than eight genes in Arabidopsis belonging to different GPAT families catalyzing either membrane or storage lipid biosynthesis have been reported. The GPAT6 falls under a different family that is involved in cutin or suberin biosynthesis in flowers and seed coats (Beisson *et al.*, 2007, 2012; Li *et al.*, 2007). *GPAT6* is strongly expressed in flowers, and *gpat6* mutants are substantially reduced in all C16 cutin monomers (DCA, 16-hydroxy- and 10,16-dihydroxypalmitates), while over-expression of *GPAT6* increased these monomers (Beisson *et al.*, 2012). After cutin monomers are synthesized, they are exported across the endoplasmic reticulum (ER) and the plasma membrane, through the polysaccharide cell wall to the nascent cuticular membrane using ATP-binding cassette (ATP) transporters. In Arabidopsis, the *CER5*/ABC transporter ABC transporter G family (ABCG) member 12 (AT1G51500) encodes an ABC transporter that is involved in cuticular wax biosynthesis (Kunst and Samuels, 2009). Recently, an ABCG transporter that played an important role in cuticle deposition was reported in wild barley and rice. A spontaneous mutation, *eibi1.b*, in wild barley led to reduced capacity to retain leaf water, a phenotype associated with reduced cutin deposition and a thin cuticle (Chen *et al.*, 2011).

Using Arabidopsis model studies the barley genes were predicted and used for expression analysis using qPCR. Our results suggested upregulation of *KAS2*, *CYP86A2*, *CYP89A2* and *LACS2* (Fig. 2), which have important roles in cuticle biosynthesis. Transcriptional regulation of cuticle biosynthesis has mainly been explored in model plants like Arabidopsis, however, in crops such as barley and wheat, information is lacking. Regulation of cuticle biosynthesis is intricate, involves a complex network of genes, hormones and TFs, and is influenced by various factors like environment, plant development and pathogen attack (Yeats and Rose, 2013). *WIN1* was the first TF that was identified in having a role in cutin biosynthesis. Overexpression of *WIN1/SHN1* led to an altered cutin composition, while silencing led to reduced cutin deposition and affected water permeability in Arabidopsis (Kannangara *et al.*, 2007). Silencing of SlSHN3 in tomato resulted in morphological alterations of the fruit epidermis and significant reduction in cuticular lipids. It was demonstrated that SlSHN3 activity is mediated by control of genes associated with cutin metabolism and epidermal cell patterning (Shi *et al.*, 2013). In barley, on the basis of positional cloning, a *Nud* gene on chromosome arm 7HL that has homology to the Arabidopsis *WIN1*/*SHN1* TF, was proposed to control biosynthesis of the hull (hulled caryopses have caryopses with adhering hulls at maturity, whereas the hulless or naked caryopses are free-threshing variant phenotypes) (Taketa *et al.*, 2008).

Literature-based studies coupled with our bioinformatics analysis on the protein–DNA interactions network using STITCH 4 software highlighted *WIN1* TF (Supplementary Fig. S3) as potentially having a significant role in FHB resistance. *HvWIN1* was cloned from five different barley genotypes (Supplementary Table S5), including CI9831 and H106-371, to find if there is any functional polymorphism. While the *HvWIN1* sequence was the same at coding sequence level, the susceptible genotype had an extra intron (Supplementary Fig. S4A, B). Many genes are known to differ in intron number and arrangement without affecting function. Analysis of transcript abundance of *HvWIN1* suggested enhanced gene expression for resistant genotype CI9831 upon pathogen inoculation (Fig. 4). Since similar levels of expression were detected in RM and SM samples, the presence of an extra intron does not affect constitutively the basal levels of *HvWIN1* expression. Since the absence of an intron was found in just one resistant genotype (CI9831), further studies including sequencing of *WIN1* from other resistant genotypes and their validation are needed to conclude the role of introns in gene regulation and disease resistance. The variation in gene expression could be due to a number of other reasons and is beyond the scope of this study.

Association of RR metabolites with genes is not enough to claim the role of that gene in FHB resistance, and they need to be functionally validated. To prove the role of *HvWIN1* in FHB disease resistance and transcriptional regulation of downstream target genes, we carried out transient gene silencing of *HvWIN1* based on VIGS (Fig. 5A), which is an easy and rapid knockdown technique to study gene function in plant development, biotic and abiotic stress resistance (Senthil-Kumar *et al.*, 2008; Cakir *et al.*, 2010; Ramegowda *et al.*, 2014) and has been successfully utilized in several crops like barley, wheat and potato (Scofield *et al.*, 2005; Lee *et al.*, 2012; Delventhal et al., 2014; Yogendra *et al.*, 2015*a*). In our study, silencing of *HvWIN1* in CI9831 resulted in a susceptible phenotype as evidenced by increased fungal biomass measured by increased copy number of *tri6* (Fig. 5C). Also, the transcript abundance of *HvWIN1* was significantly reduced due to knockdown of the gene (Fig. 5D). These results present compelling evidence of the involvement of *HvWIN1* in FHB disease resistance. Since the amount of FFAs like linoleate (FC=39.00–3.09) and palmitic acid (FC=6.56–1.3) was decreased dramatically, it was inferred that *HvWIN1* has a role in the regulation of fatty acid biosynthetic genes. However, transcript expression analysis of *KAS2* did not reflect the same upon silencing of *HvWIN1*. This could be due to the involvement of other fatty acid biosynthetic genes such as *acetyl-CoA carboxylase*, or other genes. A TF can bind to several downstream *R* genes and regulate biosynthesis of several RR metabolites. However, the high upregulation of *CYP86A2*, *CYP89A2* and *LACS2* upon pathogen invasion in resistant genotype CI9831 was significantly reduced upon *HvWIN1* silencing (Fig. 6), proving regulation of these genes by this TF. In one of the studies in Arabidopsis, *LACS2* was proved to be directly targeted by *WIN1* (Kannangara *et al.*, 2007). Our results suggest that these genes may be under the transcriptional control of *WIN1*. However, promoter analysis of these genes, except for *KAS2* to which promoter sequence was not available, failed to lead to any significant insight, as these promoters did not have the GCC box that is necessary for *WIN1* to bind to these promoters. This was consistent with other studies in Arabidopsis where, surprisingly, none of the promoters under *WIN1* control contained identifiable GCC boxes (Kannangara *et al.*, 2007). It seems that *HvWIN1* either controls FFA biosynthesis and cuticle biosynthesis genes using some unknown mechanism or in coordination with some other TFs that may regulate these genes (Alves *et al.*, 2014). A similar kind of conclusion has been made in another study in Arabidopsis (Kannangara *et al.*, 2007). In Arabidopsis and *Torenia fournieri*, MIXTA-like *MYB* TFs *MYB106* and *MYB16* that regulated epidermal cell morphology, were also shown to regulate cuticle development coordinately with *WIN1/SHN1* (Oshima *et al.*, 2013).

**Fig. 7.**
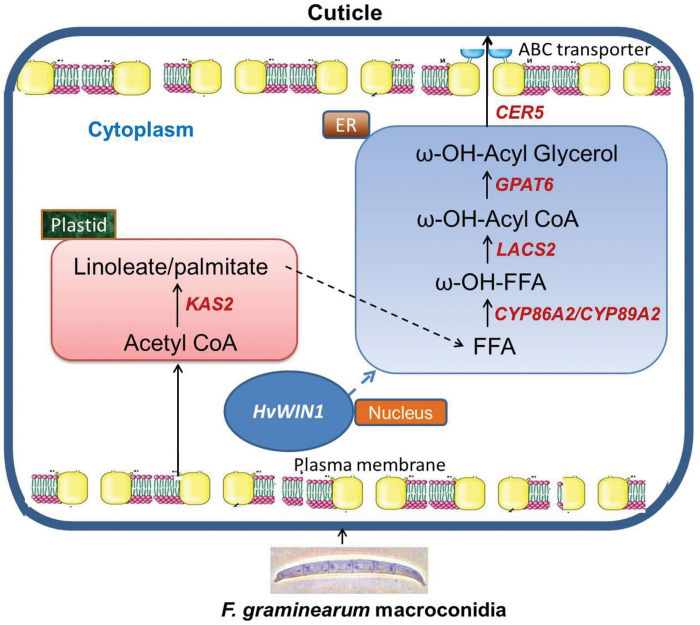
A proposed model showing *HvWIN1* regulating genes involved in cuticle biosynthesis to resist the pathogen. Genes selected for expression studies are shown in bold italics. *CER5*, *ABC transporter*; *CYP86A2*, *cytochrome P450 86A2*; *CYP89A2*, *cytochrome P450 89A2*; *GPAT6*, *glycerol-3-phosphate* acyltransferase6; KAS2, *β-ketoacyl-(acyl carrier protein) synthase II*; *LACS2*, *long-chain acyl-CoA synthetase.* A colour version of this figure is available at *JXB* online.

In conclusion, our metabolomics, gene expression and functional validation data revealed the important role of *HvWIN1* in regulating genes involved in cuticle biosynthesis. Based on our results, we have proposed a model (Fig. 7) explaining the regulation of genes involved in FFA biosynthesis by *HvWIN1* upon *F. graminearum* infection, leading to reinforcement of the cuticle. Since the germ tube of *F. graminearum* enters spikelets between the lemma and palea, gaining access through stomata on the inner side of the lemma and palea, or by direct penetration through the cuticle, the deposition of these FFAs to reinforce the cuticle can pose a physical barrier leading to disease resistance. Taken together, *HvWIN1* could be used in breeding programs or used to replace it in FHB-susceptible cultivars, if this gene is nonfunctional, based on genome editing to improve resistance in barley against FHB (Shan *et al.*, 2014).

## Supplementary data

Supplementary data are available at *JXB* online.

Fig. S1. Canonical discriminant analysis of significant (*P*<0.05) metabolites in spikelets of barley resistant (CI9831) and susceptible (H106-371) genotypes upon *F. graminearum* or mock inoculation.

Fig. S2. *In silico* fragmentation of resistance-related (RR) metabolites.

Fig. S3. Protein–DNA interactions network for *WIN1* TF based on integration of experimental and manually curated evidence with text-mining information and interaction predictions using STITCH 4 software (http://stitch.embl.de).

Fig. S4. (A) Alignment of nucleotide sequence of *HvWIN1* from CI9831 (resistant) with susceptible barley genotypes (H106-371, AC Metcalfe, CDC Copeland, Zhedar-2). (B) Alignment of amino acid sequence of coding regions of *HvWIN1* from CI9831 (resistant) and susceptible barley (H106-371, AC Metcalfe, CDC Copeland, Zhedar-2) genotypes.

Table S1. Primers used for the expression analysis of various *H. vulgare* genes involved in cuticle biosynthesis.

Table S2. BLAST analysis of *HvWIN1* fragment used for VIGS experiment.

Table S3. Resistance-related (RR) metabolites (*P*>0.05) detected in the spikelets of barley genotypes inoculated with water or spores of *F. graminearum*.

Table S4. Details of the genes predicted in barley and used in expression analysis in the present work.

Table S5. List of barley genotypes from which *HvWIN1* was sequenced and submitted to the NCBI database with GenBank accession numbers.

Table S6. Promoter analysis of potential *HvWIN1* target genes under study.

Supplementary Data
