# Supplementary material for: WAX INDUCER1 (HvWIN1) transcription factor regulates free fatty acid biosynthetic genes to reinforce cuticle to resist Fusarium head blight in barley spikelets
Source: J Exp Bot. 2016 May 18;67(14):4127–39. doi: 10.1093/jxb/erw187 (PMC5301922; doi:10.1093/jxb/erw187)
Supplement: Supplementary Data [file supp_67_14_4127__index.html]

WAX INDUCER1 (HvWIN1) transcription factor regulates free fatty acid biosynthetic genes to reinforce cuticle to resist Fusarium head blight in barley spikelets — WAX INDUCER1 (HvWIN1) transcription factor regulates free fatty acid biosynthetic genes to reinforce cuticle to resist Fusarium head blight in barley spikelets — Supplementary Data 

# WAX INDUCER1 (HvWIN1) transcription factor regulates free fatty acid biosynthetic genes to reinforce cuticle to resist Fusarium head blight in barley spikelets

## Supplementary Data

Data files

- Supplementary\_figures\_S1\_S4\_\_tables\_S1\_S6.docx - Supplementary Data
