# Supplementary material for: WAX INDUCER1 (HvWIN1) transcription factor regulates free fatty acid biosynthetic genes to reinforce cuticle to resist Fusarium head blight in barley spikelets
Source: J Exp Bot. 2016 May 18;67(14):4127–39. doi: 10.1093/jxb/erw187 (PMC5301922; doi:10.1093/jxb/erw187)
Supplement: Supplementary Data [file supp_erw187_Supplementary_figures_S1_S4__tables_S1_S6.docx]

**WAX INDUCER1 (HvWIN1) transcription factor regulates free fatty acid biosynthetic genes to enforce cuticle to resist fusarium head blight in barley spikelets**

Arun Kumar^1!^, Kalenahalli N. Yogendra^1^, Shailesh Karre^1^, Ajjamada C. Kushalappa^1*^, Yves Dion^2^, Thin M. Choo^3^


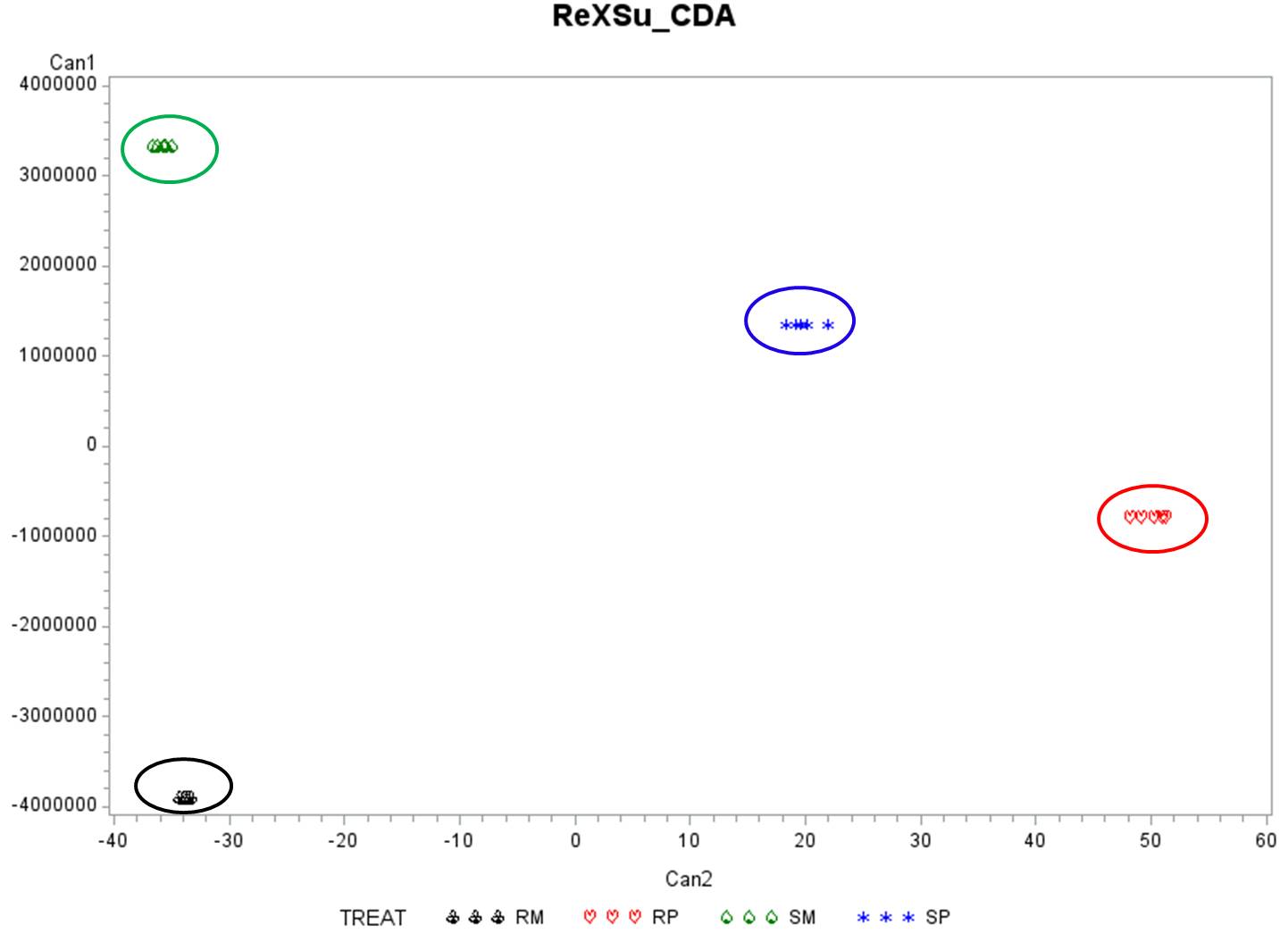


**Fig. S1.** Canonical discriminant analysis of significant (*P*<0.05) metabolites in spikelets of barley resistant (CI9831) and susceptible (H106-371) genotypes upon *F. graminearum* or mock inoculation. Where, RP is *F. graminearum* inoculated resistant genotype CI9831, RM is mock-inoculated resistant genotype CI9831, SP is *F. graminearum* inoculated susceptible genotype H106-371, SM is mock-inoculated susceptible genotype H106-371.


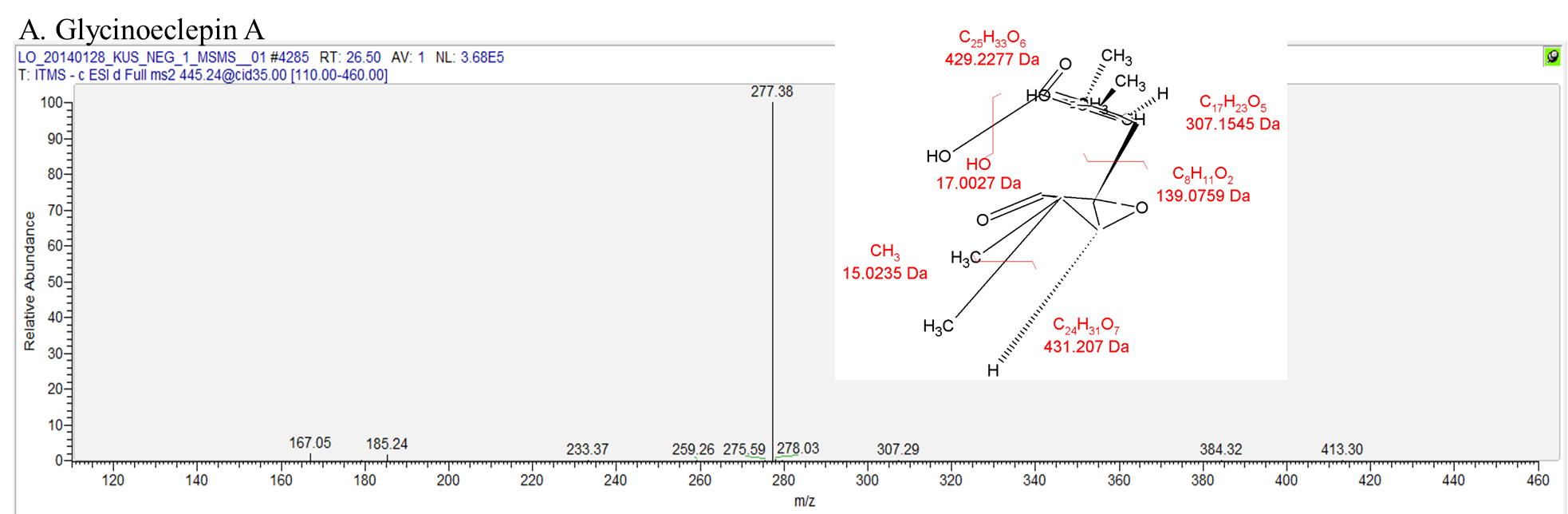


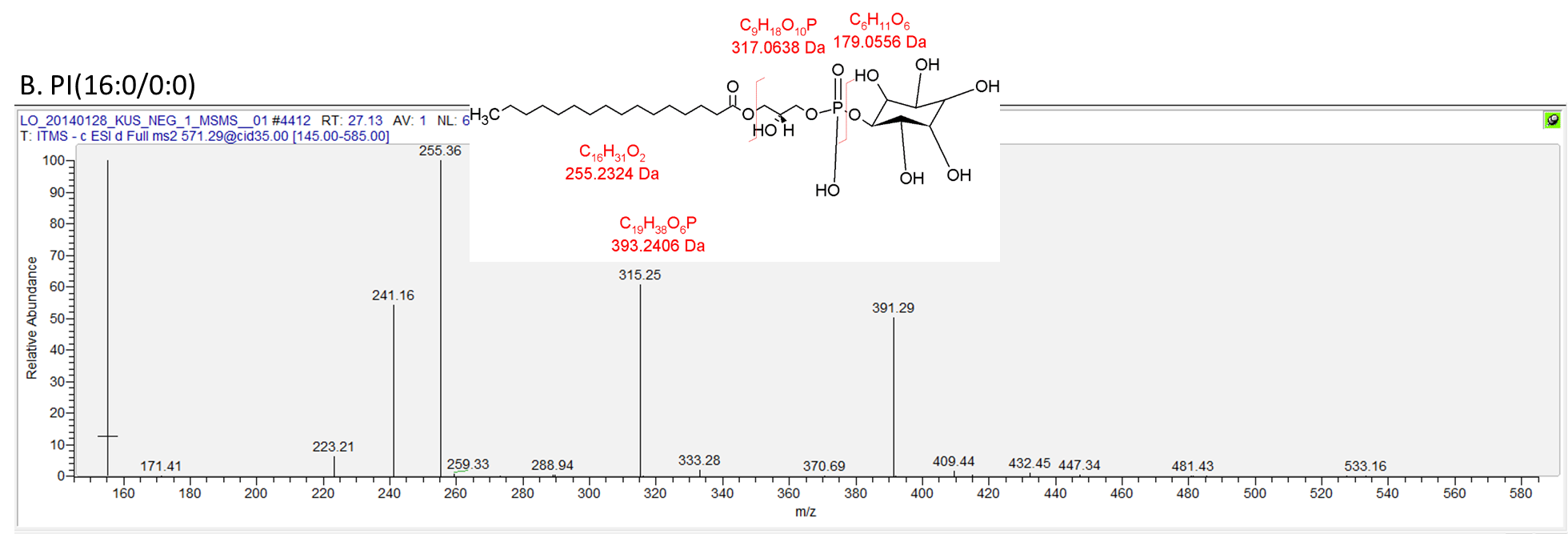


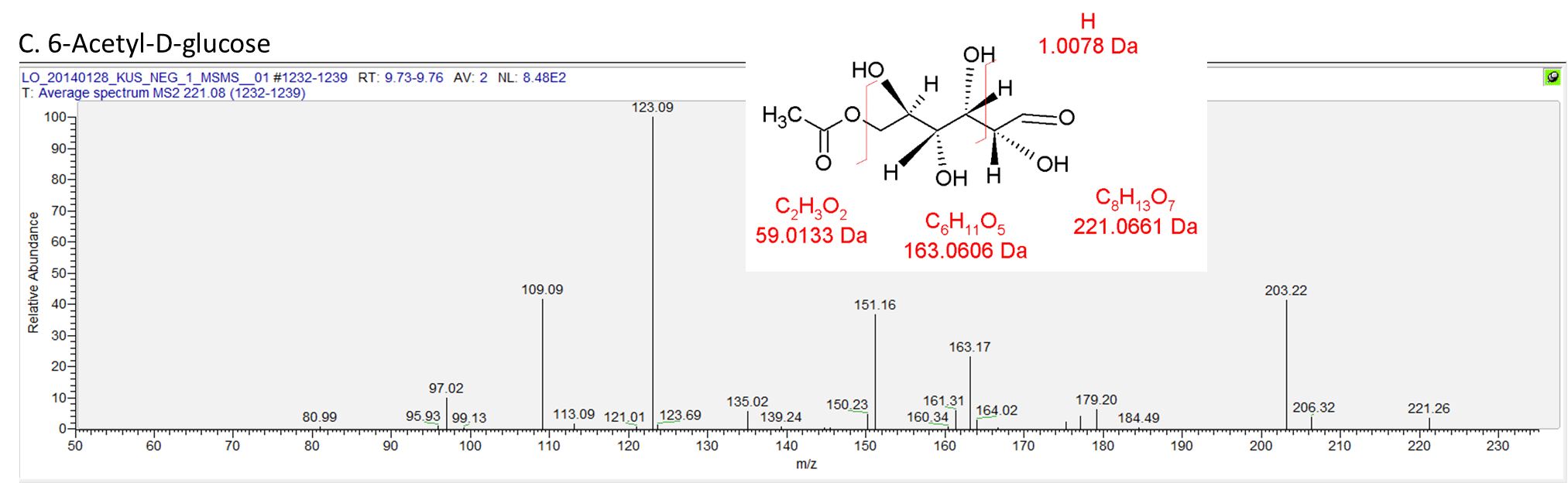


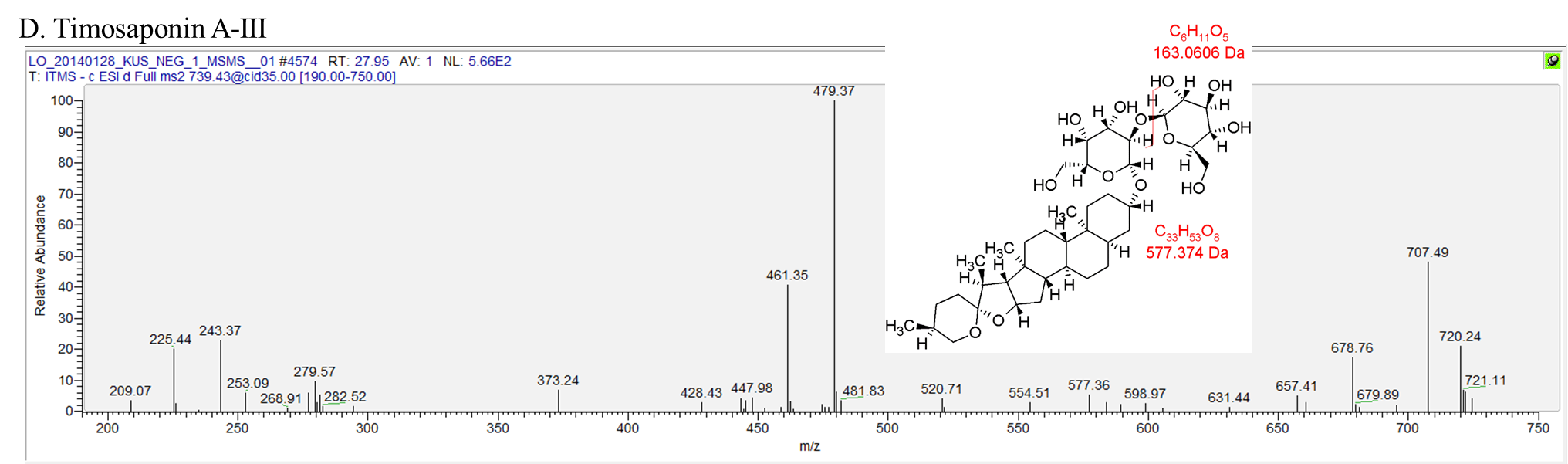


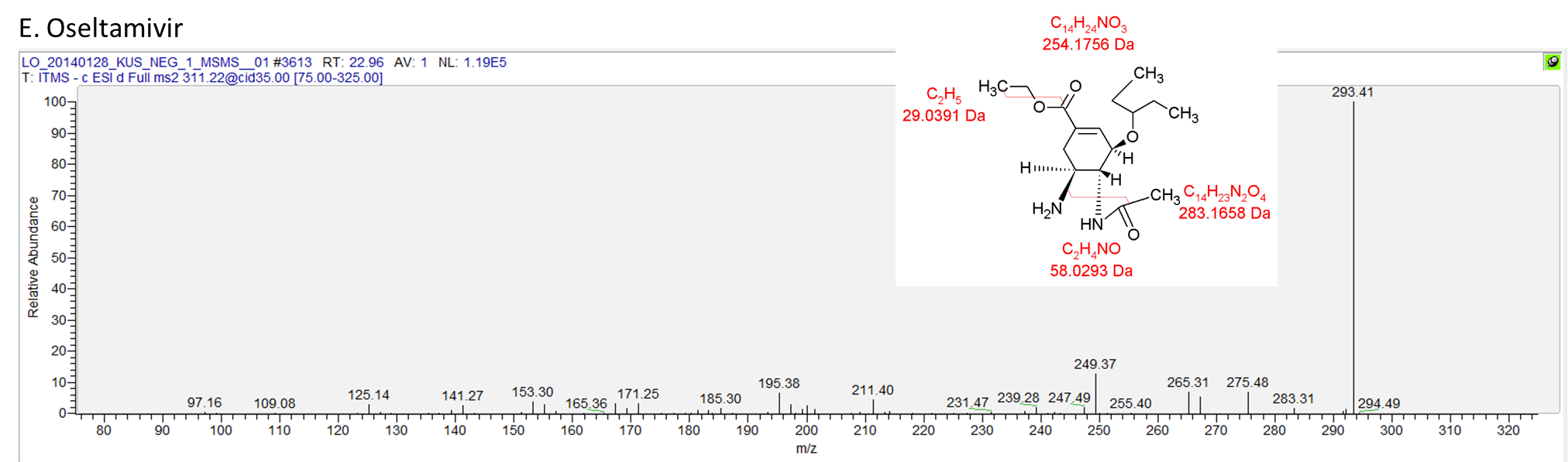


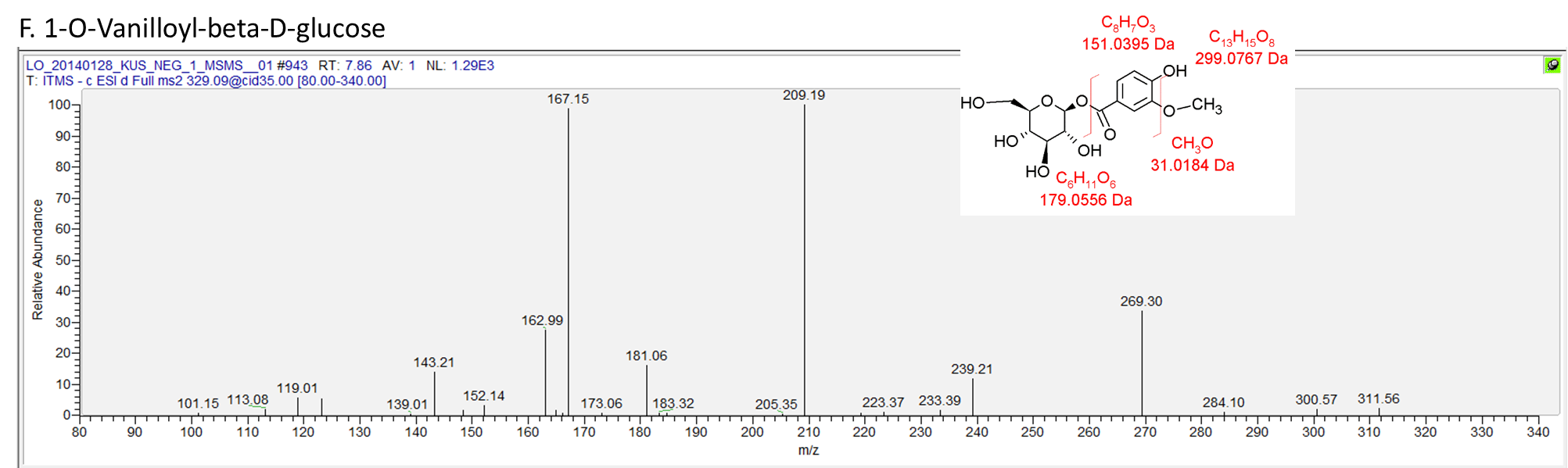


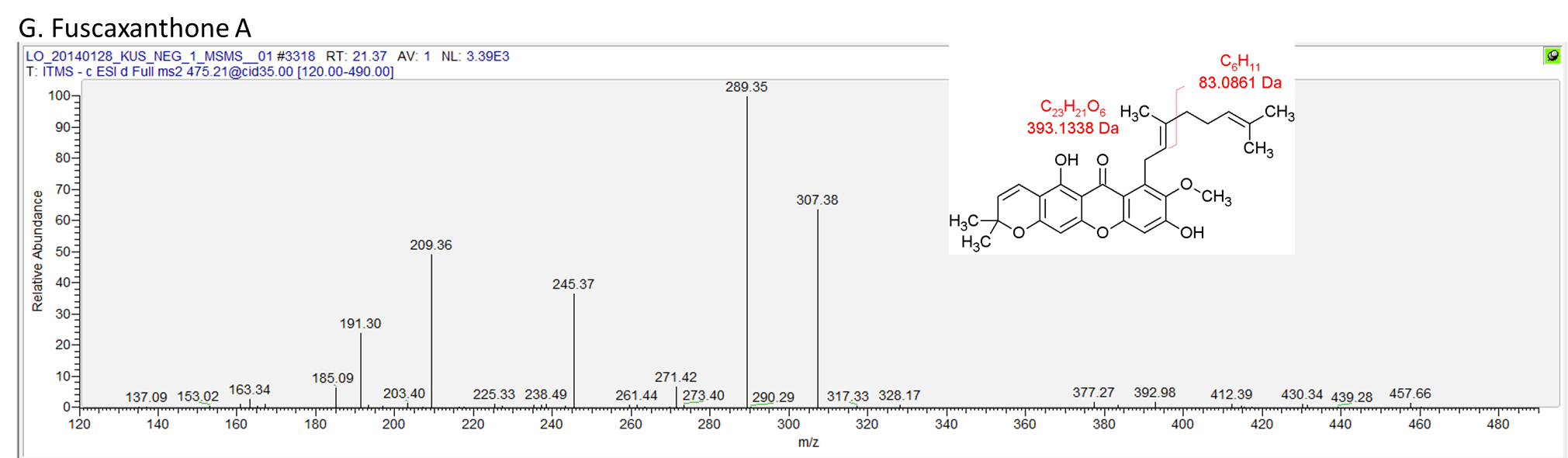


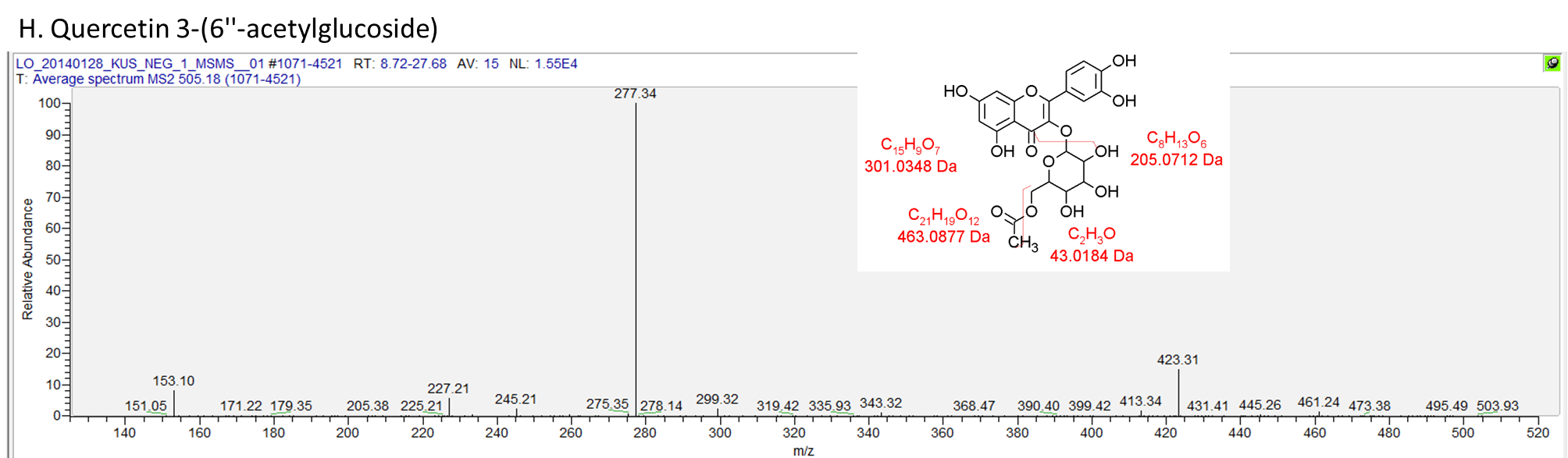


**Fig. S2. *In silico* fragmentation of resistance related metabolites.**


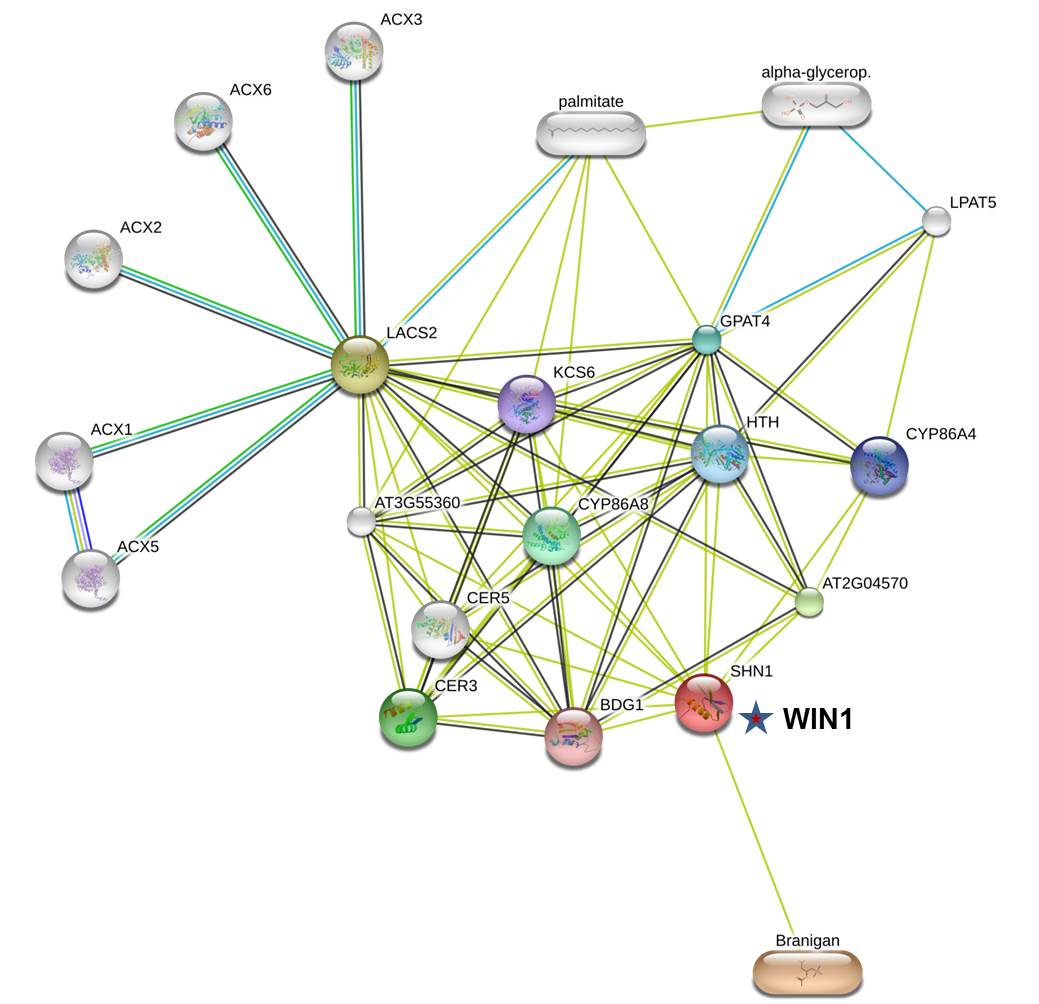


**Fig. S3.** Protein–DNA interactions network for *WIN1* TF based on integration of experimental and manually curated evidence with text-mining information and interaction predictions using STITCH 4 software [(http://stitch.embl.de](http://stitch.embl.de/)). The *WIN1* transcription factor is shown with an asterisk mark, which is shown as SHN1 in figure.

**
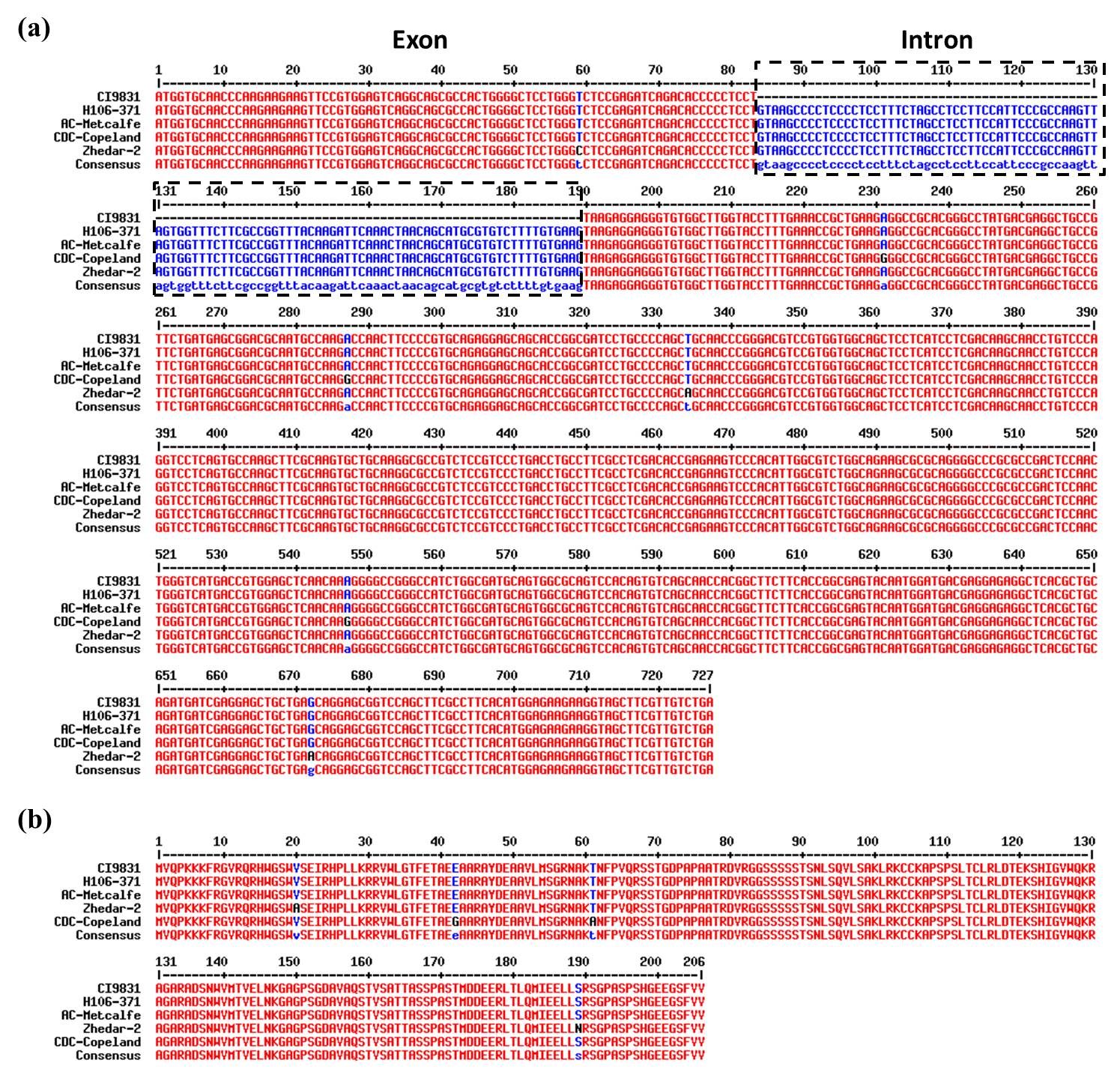
**

**Fig. S4.** (a) Alignment of nucleotide sequence of *HvWIN1* from CI9831 with susceptible barley genotypes (H106-371, AC Metcalfe, CDC Copeland, Zhedar-2). Nucleotides in red color represent the coding region of *HvWIN1* whereas, nucleotides in black color (inside bracket) represent the intron region of *HvWIN1* which is absent in CI9831. (b) Alignment of amino acid sequence of coding region of *HvWIN1* from CI9831 (resistant) with susceptible barley genotypes (H106-371, AC Metcalfe, CDC Copeland, Zhedar-2).

### Table S1: Primers used for expression analysis of various *H. vulgare* genes involved in cuticle biosynthesis.*Actin*, *β-Actin*; *KAS2*, β-ketoacyl-(acyl carrier protein) synthase II; *CYP86A2*, *cytochrome P450 86A2*; *CYP89A2*, *cytochrome P450 89A2*; *LACS2*, *long-chain acyl-CoA synthetase*; *GPAT6*, *glycerol-3-phosphate acyltransferase6*; *CER5*, *ABC transporter.*

| **S. No.** | **Gene** | **Forward Primer (5’-3’)** | **Reverse Primer (5’-3’)** | **Amplicon size (bp)** |
| --- | --- | --- | --- | --- |
| 1 | *Actin* | GGAATCCACGAGACGACCTACA | CTTGCTCATACGGTCAGCGATA | 129 |
| 3 | *KAS2* | TATTCCGATTGGTGTTGGTG | CCTTCACCCATAACGAAACC | 120 |
| 4 | *CYP86A2* | ACCTGCAGATGAAGAACATC | TACAAGTTTCCTCCATCCGT | 135 |
| 5 | *CYP89A2* | GCAACCGAGAGATCAAGATG | GCCACTCGAACTCCTTTACC | 120 |
| 6 | *LACS2* | TATGGGCTCACTGAGAGTTG | ACCCATTTCAGGAACAGACT | 126 |
| 7 | *GPAT6* | TGACGTCGAGGAAGTACAGC | ATCCAGAGGAAGGTGACGAG | 121 |
| 8 | *CER5* | TTCGCCCTTAACCTCTATGG | ACATCATGATCCCAATGACG | 124 |

**Table S2:** BLAST analysis of *HvWIN1* fragment used for VIGS experiment. The nucleotide sequence of *HvWIN1* (250 bp) used for VIGS experiment was subjected to blast analysis in IPK Barley BLAST server (<http://webblast.ipk-gatersleben.de/barley/>). The blast analysis showed maximum homology to [morex_contig_1564026](http://apex.ipk-gatersleben.de/apex/f?p=284:24:::NO::P24_MOREX_CONTIG:morex_contig_1564026) that encodes *HvWIN1*.

| **Query** | **Subject** | **Score** | **Identities (query length)** | **Expect** |
| --- | --- | --- | --- | --- |
| Query1 | morex_contig_1564026 | 394 | 220/221 (250) | 7e-108 |
| Query1 | morex_contig_43456 | 143 | 177/242 (250) | 2e-32 |
| Query1 | morex_contig_63458 | 59.0 | 81/110 (250) | 7e-07 |
| Query1 | morex_contig_45749 | 53.6 | 43/52 (250) | 3e-05 |
| Query1 | morex_contig_1565327 | 50.0 | 71/100 (250) | 4e-04 |
| Query1 | morex_contig_46805 | 50.0 | 45/57 (250) | 4e-04 |
| Query1 | morex_contig_1646865 | 46.4 | 46/60 (250) | 0.004 |
| Query1 | morex_contig_1637676 | 46.4 | 39/48 (250) | 0.004 |
| Query1 | morex_contig_350984 | 46.4 | 75/108 (250) | 0.004 |
| Query1 | morex_contig_161630 | 46.4 | 80/113 (250) | 0.004 |
| Query1 | morex_contig_1819680 | 44.6 | 30/33 (250) | 0.015 |
| Query1 | morex_contig_1716014 | 44.6 | 29/31 (250) | 0.015 |
| Query1 | morex_contig_137502 | 44.6 | 75/109 (250) | 0.015 |

**Table S3.** Resistance related (RR) metabolites (p>0.05) detected in the spikelets of barley genotype CI9831 relative to a susceptible genotype H106-371, in which spikelets were inoculated with water or spores of *F. graminearum*. The details about fragmentation pattern of important metabolites are also provided. AME: Accurate Mass Error = ((Observed mass - expected mass) / expected mass) × 10^6^, RT: Retention time, RRC: Resistance related constitutive, RRI: Resistance related induced metabolites. Fold change calculation: were based on relative intensity of metabolites, RRC= RM/SM, RRI= (RP/RM)/ (SP/SM); RRI= RP/RM. RP: Resistant cultivar with pathogen inoculation, RM: Resistant cultivar with mock inoculation, SP: Susceptible cultivar with pathogen inoculation, SM: Susceptible cultivar with mock inoculation.

Database ID examples: Number-METLIN, LMP-LIPIDMAPS, KEGG-C05610, KNAPSACK- C00002775, and PMN-Plant Metabolic Network, *In silico*: In silico fragmentation.

| **Observed mass (Da)** | **Exact mass (Da)** | **AME** | **RT (min)** | **Putative Name** | **Chemical group** | **Observed fragmentation** | **Database Fragmentation** | **P value** | **FC** | **Database ID** |
| --- | --- | --- | --- | --- | --- | --- | --- | --- | --- | --- |
| 280.2405 | 280.2400 | 1.05 | 27.33 | Linoleate 9(E),11(E)  -Conjugated Linoleic Acid | Fatty acids | **96.96,206.34,** 219.40, | **96.95**, **206.16**, 231.14 | 0.0072 | 38.99 (RRI) | MID: 34793 |
| 449.2769 | 449.2800 | -1.75 | 27.31 | Condelphine | diterpenoid alkaloids |  | No database fragmentation | 0.0033 | 22.99 (RRI) | C08671 |
| 446.2295 | 446.2300 | -2.28 | 26.49 | Glycinoeclepin A | Terpenoid | 279.50, **307.29**, 384.32 | **307.1545** | 0.0492 | 7.73 RRI | C08765 |
|  |  |  |  |  |  |  |  |  |  |  |
| 256.2406 | 256.2400 | 1.52 | 29.28 | Hexadecanoic acid | Fatty acids | 111.21, 139.29, 179.26, **237.38**, **255.39** | **237.22**, **255.23** | 0.0365 | 6.56 RRI | C00249 MID:187 |
|  |  |  |  |  |  |  |  |  |  |  |
| 572.2962 | 572.2960 | -0.07 | 27.17 | PI(16:0/0:0) | Glycerophospholipids | 171.41, 223.21, 241.16, **255.36**, 315.25, 391.29 | 179.0556, **255.2324**, 317.0638, 393.2406 | 0.0184 | 4.21 (RRI) | LMGP06050002 |
|  |  |  |  |  |  |  |  |  |  |  |
| 596.3534 | 596.3500 | 3.15 | 29.07 | C-Curarine | Alkaloids |  |  | 0.0379 | 3.26 (RRI) | C09144 |
| 870.4980 | 870.4977 | 0.32 | 27.71 | Racemoside C | Steroidal saponin | 506.22, 517.95, 567.06, 725.44, 771.98 | No database fragmentation | 0.0128 | 3.12 RRI | 905929-91-1 |
|  |  |  |  |  |  |  |  |  |  |  |
| 202.1211 | 202.1200 | 2.75 | 19.85 | Sebacic acid | Fatty acid | **137.35**, **139.40**, 155.00, **157.27**, 169.36, **183.13** | **137.09**, **139.11**, **157.12**, **183.10,** 201.11 | 0.0233 | 2.79 RRI | C08277 |
| 222.0743 | 222.0700 | 1.36 | 0.85 | 6-Acetyl-D-glucose | Carbohydrate derivative | **221.26,** 203.22, **163.17**, 123.09 | **221.06, 163.06** | 0.0242 | 2.72 RRI | C02655 |
| 450.3218 | 450.3220 | -1.06 | 27.97 | C17 sphingosine-1-phosphocholine | Sphingolipid |  |  | 0.0263 | 2.69 (RRI) | LMSP01060003 |
| 424.2723 | 424.2700 | -0.61 | 27.64 | DevapamilArachidonoyl m-Nitroaniline |  | **275.23,** 341.25, 361.30, 390.49 | **275.13**, 297.13, 311.14, 423.26, 424.12 | 0.0079 | 2.60 (RRI) | C13763 **MID: 96559** |
| 386.2669 | 386.2700 | 0.23 | 27.21 | 6-Deoxyerythronolide B | Polyketides |  |  | 0.0093 | 2.51 (RRI) | C03240 |
| 326.2005 | 326.2000 | 3.40 | 26.53 | Ajmaline | Alkaloids (derived from tryptophan and anthranilic acid) | **212.19**, 293.49, **309.56**, | **212.10,** 238.12, **309.19**, 327.20 | 0.0142 | 2.44 (RRI) | C06542 |
| 410.2435 | 410.2400 | 0.50 | 26.80 | 1Palmitoyl glycerol 3 phosphate | Glycerophospholipids | 167.18, 185.39, 213.29, 241.21, 267.33, 294.24, 327.17, 351.21, 364.44, 391.87 | 78.95, 96.96, 152.99, 153.13, 171.00, 255.23, 409.24 | 0.0163 | 2.43 (RRI) | C04036 |
| 608.5013 | 608.5016 | -0.51 | 29.38 | 4-Deoxyannomontacin | mono-tetrahydrofuran (THF) gamma-lactone |  |  | 0.0252 | 2.34 (RRI) | 237762-43-5 |
| 596.2439 | 596.2410 | 4.85 | 1.66 | Magnolignan G | Lignan |  |  | 0.0016 | 2.29 (RRI) | 138581-11-4 |
| 372.1212 | 372.1200 | 0.78 | 16.89 | Sinensetin | Flavonoid | **151.12**, 160.20, **175.11**, , **278.36**, **321.49**, 327.18, | **151.04,175.04**, **278.05**, **327.06**, 329.10 | 0.0445 | 2.20 (RRI) | C10186 |
| 596.3717 | 596.3713 | 0.61 | 33.27 | Methylanhydrovilangin | benzoquinone derivatives |  |  | 0.0387 | 2.19 (RRI) | 14547-59-2 |
| 312.2048 | 312.2000 | -0.30 | 22.97 | Oseltamivir | Seems a synthetic compound | 294.49,293.41,**283.31**,**255.40**,249.67 | **283.16,254.17** | 0.0403 | 2.15 (RRI) | C08092 |
| 424.2459 | 424.2500 | -0.40 | 27.62 | Pravastatin | Fatty acid: Seems like a fungal compound | 153.08, 167.07, 185.09, 237.49, 253.64, 255.30, | 41.04, 43.01, 59.01, 59.08, 85.02, 101.06, 127.05, 143.08 | 0.0081 | 2.13 (RRI) | C01844 |
| 340.2158 | 340.2151 | 2.15 | 21.85 | (+)-Sandwicolidine | dihydroindole alkaloid | 261.30, 267.17, 281.36, 293.49, 309.56, 325.21, 326.30 | No database fragmentation | 0.039 | 2.10 (RRI) | 99612-65-4 |
| 622.4442 | 622.4445 | -0.51 | 27.86 | 20(R)-Ginsenoside Rh2 | Plant glycoside |  |  | 0.0293 | 2.09 (RRI) | 112246-15-8 |
| 740.4347 | 740.4300 | 0.01 | 27.99 | Timosaponin A-III | Saponin | 707.49, **577.36,** 479.37,461.35 | **577.37**, 163.06 | 0.009 | 2.08 (RRI) | C17075 |
| 324.2674 | 324.2660 | 3.16 | 23.47 | Auricolic acid | Fatty acids |  |  | 0.0253 | 2.05 (RRI) | LMFA01050431 |
| 868.5171 | 868.5184 | -1.55 | 28.71 | Nephelioside I | Saponin |  |  | 0.0455 | 2.04 (RRI) | 656253-22-4 |
| 270.0532 | 270.0528 | 1.43 | 16.76 | Apigenin | Flavonoid | **149.16,159.13**, **183.16**, **201.18**, **225.22**, **227.10** | **149.02**, **159.04**, **183.04**, **201.05**, **225.05**, **269.04** | 0.0014 | 2.45 (RRC) | C01477 **MID:3397** |
| 330.0951 | 330.0951 | -0.02 | 7.94 | 1-O-Vanilloyl-beta-D-glucose | *O*-acyl carbohydrate | **300.57,**209.19,**181.06**,167.15,162.99,143.21 | **299.07,179.05**,151.03,31.01 | 0.0458 | 3.36 (RRC) | C20470 |
| 338.1165 | 338.1154 | 3.19 | 14.78 | (-)-Glyceollin I Demethoxycurcumin | Phytoalexin (prenylatedpterocarpan) | **191.21**, 207.22, **217.23**, 223.32, 233.26 | 187.03, **191.07**, 201.01, 202.02, **217.05** | 0.0116 | 5.50 (RRC) | C01701 **MID: 64196** |
| 360.1057 | 360.1056 | 0.16 | 9.31 | Syringic acid beta-glucopyranosyl ester | Phenylpropanoid | 197.19, 209.23, 211.10, 224.25, 239.20, 251.22, | No database fragmentation | 0.0345 | 3.50 (RRC) | 112667-09-1 |
| 390.1893 | 390.1890 | 0.87 | 11.97 | (-)-11-hydroxy-9,10-dihydrojasmonic acid 11-beta-D-glucoside | Fatty acids | 178.91, **209.25**, 221.23, 223.19, 235.43, 243.33, **259.37,** 270.29, | 198.12, **209.09**, 226.11, 230.12, 231.11, 232.09, 244.12, **259.10,** | <.0001 | 2.75 (RRC) | LMFA02020014 |
| 418.1631 | 418.1628 | 0.79 | 15.52 | Euparotin acetate (It can be Lirioresinol A, (+)-Syringaresinol) | Terpenoids | 258.29, 303.26, 319.31, 335.27, 367.08, 371.28, | No database fragmentation available | 0.0422 | 2.15 (RRC) | C09435 |
| 432.1061 | 432.1056 | 1.22 | 15.52 | VitexinPelargonidin 3-O-glucoside | Flavonoid | 257.23, **269.17**, **283.45**, 295.34, **311.19**, 321.57 | 268.03, **269.04**, 271.05, **283.05**, **311.06**, 431.10 | 0.0187 | 2.91 **(RRC)** | C01460 **MID: 64247** |
| 506.1062 | 506.1060 | 0.49 | 14.31 | Quercetin 3-(6''-acetylglucoside) | Flavonoid | **461.24**,423.31,**299.32**,277.34,227.21,**205.38** | **463.08, 301.03,205.07** | <.0001 | 2.96 (RRC) | LMPK12110566 |
| 578.1643 | 578.1636 | 1.14 | 16.52 | ViolanthinRhoifolin | Flavonoid | 186.21, 222.11, 253.23, **269.19**, **413.35**, 465.25, | 268.03, **269.04**, 270.04, **413.09**, 577.16, 577.94 | 0.0018 | 12.11 (RRC) | C10196 **MID: 44401** |
| 596.1728 | 596.1741 | -2.13 | 12.76 | Isobutrin | Flavonoid |  | No database fragmentation available | 0.0081 | 2.04 (RRC) | C08649 |
| 596.3545 | 596.3515 | 5.02 | 3.83 | C-Curarine | Alkaloid | 271.17, 293.15, 311.24, 325.08, |  | 0.0031 | 2.02 (RRC) | C09144 |
| 608.2114 | 608.2105 | 4.30 | 15.55 | Matteucinol 7-O-beta-D-apiofuranosyl(1->6)-beta-D-glucopyranoside | Flavonoid | 220.68, 247.36, 305.31, 336.33, 352.09, 355.17, | No database fragmentation available | 0.0069 | 2.22 (RRC) | LMPK12140329 |
| 610.3844 | 610.3846 | -0.39 | 29.08 | PG(12:0/12:0) | Glycerophospholipid | **593.43**, 591.43, **534.38,** 523.36, | **593.38, 535.34** | 0.0097 | 2.73 (RRC) | LMGP04010035 |
| 732.4579 | 732.4601 | -2.99 | 29.59 | 3-(E)-Coumaroylbetulin-28-yl-ethyl (2R)-2-hydroxysuccinate | Terpenoid | 209.36, 245.31, | No database fragmentation available | 0.0391 | 2.68 (RRC) | 302944-08-7 |
| 740.2163 | 740.2164 | -0.16 | 16.40 | Robinin (kaempferol-3-O-robinoside-7-O-rhamnoside) | Flavonoid | 225.08, 252.20, 269.22, 281.31, 311.30, 324.18, | 256.00, 376.17, 392.20, 404.24, | 0.0038 | 4.84 (RRC) | C10178 |
| 740.4632 | 740.4628 | 0.53 | 29.51 | PG(14:1(9Z)/20:4(5Z,8Z,11Z,14Z)) | Glycerophospholipid | 477.16, 479.28, 511.92, 544.12, 576.41, 640.95, | No database fragmentation | 0.0318 | 5.44 (RRC) | LMGP04010131 |
| 748.2949 | 748.2942 | 0.91 | 16.02 | Alangisesquin C | Lignan | 551.25, 585.22, 606.49, 634.04, 667.31, 699.17, | No database fragmentation | 0.0043 | 4.52 (RRC) | 212007-44-8 |
| 754.4414 | 754.4414 | 1.30 | 29.05 | PC(5:0/30:11) | Glycerophospholipid | 475.36, 493.36, 517.44, 553.35, 571.61, 589.16, 615.46, 641.39, | No database fragmentation | 0.0063 | 3.32 (RRC) |  |
| 772.4522 | 772.4522 | 1.50 | 28.37 | PS(0:0/37:10) | Glycerophospholipid | 429.36, 461.20, 477.59, 495.15, 516.58, 542.08, |  | 0.0317 | 2.66 (RRC) |  |
| 788.4469 | 788.4476 | 2.00 | 27.63 | PI(13:0/18:4(6Z,9Z,12Z,15Z)) | Glycerophospholipid | 479.33, 521.35, 577.08, 618.38, 625.94, 654.29, |  | 0.0025 | 2.04 (RRC) | LMGP06010049 |
| 790.4656 | 790.4632 | 3.07 | 27.70 | PI(13:0/18:3(6Z,9Z,12Z)) | Glycerophospholipid | 477.43, 495.33, 550.58, 579.18, 593.58, 627.20, |  | 0.01 | 2.07 (RRC) | LMGP06010047 |
| 956.8784 | 956.8772 | 3.00 | 25.84 | TG(19:0/20:1(11Z)/20:1(11Z))[iso3] | Glycerophospholipid | 866.28, 871.51, 911.67, 926.44, 940.76 |  | 0.0272 | 2.83 (RRC) | LMGL03011003 |

**Table S4.** Table showing details of the genes predicted barley and used in expression analysis in the present work.*KAS2*, β-ketoacyl-(acyl carrier protein) synthase II; *CYP86A2*, *cytochrome P450 86A2*; *CYP89A2*, *cytochrome P450 89A2*; *LACS2*, *long-chain acyl-CoA synthetase*; *GPAT6*, *glycerol-3-phosphate acyltransferase6*; *CER5*, *ABC transporter.*

| **S. No.** | **Gene** | **morex_contig** | **Chromosome** | **Coding sequence (bp)** | **Predicted protein (aa)** |
| --- | --- | --- | --- | --- | --- |
| 1 | *KAS2* | 48560 | 7HS | 1227 | 408 |
| 2 | *CYP86A2* | 66967 | 6HL | 1539 | 512 |
| 3 | *CYP89A2* | 1558559 | 1H | 1551 | 517 |
| 4 | *LACS2* | 52159 | 5HL | 1491 | 496 |
| 5 | *GPAT6* | 40562 | 3HL | 984 | 327 |
| 6 | *CER5* | 1559109 | 1H | 2352 | 783 |
| 7 | *HvWIN1* | 1564026 | 6HS | 621 | 206 |

**Table S5.** List of barley genotypes from which *HvWIN1* was sequenced and submitted to NCBI database with their GenBank accession numbers.

| **Genotype** | **GenBank accession no.** | **CDS (bp)** |
| --- | --- | --- |
| CI9831 | KT946819 | 621 |
| H106-371 | KT946820 | 621 |
| Zhedar-2 | KT946821 | 621 |
| AC-Metcalfe | KT946822 | 621 |
| CDC Copeland | KT946823 | 621 |

**Table S6.** Promoter analysis of potential *HvWIN1* target genes under study. Promoter sequences were analyzed using PlantCare software.

| ***CYP86A2*** | ***CYP89A2*** | ***LACS2*** | ***GPAT6*** |
| --- | --- | --- | --- |
| A-box: cis-acting regulatory element | AAGAA-motif : cis-acting element conferring high transcription levels | A-box: cis-acting regulatory element | ABRE: cis-acting element involved in the abscisic acid responsiveness |
| ABRE: cis-acting element involved in the abscisic acid responsiveness | ABRE: cis-acting element involved in the abscisic acid responsiveness | ABRE: cis-acting element involved in the abscisic acid responsiveness | ARE: cis-acting regulatory element essential for the anaerobic induction |
| ARE: cis-acting regulatory element essential for the anaerobic induction | CAAT-box: common cis-acting element in promoter and enhancer regions | ATGCAAAT motif: cis-acting regulatory element associated to the TGAGTCA motif | Box-W1: fungal elicitor responsive element |
| CAAT-box: common cis-acting element in promoter and enhancer regions | G-Box: cis-acting regulatory element involved in light responsiveness | Box II: part of a light responsive element | CAAT box: common cis-acting element in promoter and enhancer regions |
| CCGTCC-box: cis-acting regulatory element related to meristem specific activation | GT1-motif: cis-acting regulatory element involved in light responsiveness | CAAT-box: common cis-acting element in promoter and enhancer regions | CATT motif: MYBHv1 binding site |
| CE3: cis-acting element involved in ABA and VP1 responsiveness | G-box: light responsive element | CATT-motif: part of a light responsive element | CCAAT-box: cis-acting regulatory element involved in the MeJA-responsiveness |
| CTAG-motif:-- | Skn-1_motif: cis-acting regulatory element required for endosperm expression | CCAAT-box: MYBHv1 binding site | CGTCA-motif: cis-acting regulatory element involved in light responsiveness |
| G-Box: cis-acting regulatory element involved in light responsiveness | TATA-box: core promoter element around -30 of transcription start | CCGTCC-box: cis-acting regulatory element related to meristem specific activation | G-box: cis-acting regulatory element involved in light responsiveness |
| Gap-box: m part of a light responsive element | TCA-element: cis-acting element involved in salicylic acid responsiveness | G-Box: cis-acting regulatory element involved in light responsiveness | GARE-motif: gibberellin-responsive element |
| MBS: MYB binding site involved in drought-inducibility | TCT-motif: part of a light responsive element | GAG-motif: part of a light responsive element | GC-motif: enhancer-like element involved in anoxic specific inducibility |
| RY-element: cis-acting regulatory element involved in seed-specific regulation | as-2-box: involved in shoot-specific expression and light responsiveness | GCN4_motif : cis-regulatory element involved in endosperm expression | MBS: MYB binding site involved in drought-inducibility |
| Sp1: light responsive element | Circadian: cis-acting regulatory element involved in circadian control | HSE: cis-acting element involved in heat stress responsiveness | MNF1: light responsive element |
| TATA-box: core promoter element around -30 of transcription start |  | LTR: cis-acting element involved in low-temperature responsiveness | Skn-1 motif: cis-acting regulatory element required for endosperm expression |
| TGA-element: auxin-responsive element |  | MBS: MYB binding site involved in drought-inducibility | Sp1: light responsive element |
| TGG-motif: part of a light responsive element |  | MNF: light responsive element | TATA box: core promoter element around -30 of transcription start |
| plant_AP-2-like motif: Can be important in disease resistance |  | MSA –like: cis-acting element involved in cell cycle regulation | TATCCAT/C-motif: |
|  |  | O2-site: cis-acting regulatory element involved in zein metabolism regulation | TGACG-motif: cis-acting regulatory element involved in the MeJA-responsiveness |
|  |  | Skn-1_motif : cis-acting regulatory element required for endosperm expression | W box |
|  |  | Sp1: light responsive element | chs-CMA2a: part of a light responsive element |
|  |  | TATA-box: core promoter element around -30 of transcription start | Circadian: part of a light responsive element |
|  |  | TCA-element: cis-acting element involved in salicylic acid responsiveness |  |
|  |  | TGA-element: auxin-responsive element |  |
